# TBP and SNAP50 transcription factors bind specifically to the Pr77 promoter sequence from trypanosomatid non-LTR retrotransposons

**DOI:** 10.1186/s13071-021-04803-5

**Published:** 2021-06-09

**Authors:** Francisco Macías, Raquel Afonso-Lehmann, Patricia E. Carreira, M. Carmen Thomas

**Affiliations:** 1 Departamento de Biología Molecular, Instituto de Parasitología y Biomedicina “López Neyra”, Consejo Superior de Investigaciones Científicas, Parque Tecnológico de Ciencias de la Salud, 18016 Granada, Spain; 2 Present Address: Mater Research Institute, University of Queensland, TRI Building, Woolloongabba, QLD 4102 Australia

**Keywords:** Trypanosomatid, Non-LTR retrotransposon, L1Tc, NARTc, Transcription, Promoter, Downstream promoter element (DPE)

## Abstract

**Background:**

Trypanosomatid genomes are colonized by active and inactive mobile DNA elements, such as LINE, SINE-like, SIDER and DIRE retrotransposons. These elements all share a 77-nucleotide-long sequence at their 5′ ends, known as Pr77, which activates transcription, thereby generating abundant unspliced and translatable transcripts. However, transcription factors that mediates this process have still not been reported.

**Methods:**

TATA-binding protein (TBP) and small nuclear RNA-activating protein 50 kDa (SNAP50) recombinant proteins and specific antibodies raised against them were generated. Protein capture assay, electrophoretic mobility-shift assays (EMSA) and EMSA competition assays carried out using these proteins and nuclear proteins of the parasite together to specific DNA sequences used as probes allowed detecting direct interaction of these transcription factors to Pr77 sequence.

**Results:**

This study identified TBP and SNAP50 as part of the DNA-protein complex formed by the Pr77 promoter sequence and nuclear proteins of *Trypanosoma cruzi*. TBP establishes direct and specific contact with the Pr77 sequence, where the DPE and DPE downstream regions are docking sites with preferential binding. TBP binds cooperatively (Hill coefficient = 1.67) to Pr77 and to both strands of the Pr77 sequence, while the conformation of this highly structured sequence is not involved in TBP binding. Direct binding of SNAP50 to the Pr77 sequence is weak and may be mediated by protein–protein interactions through other trypanosomatid nuclear proteins.

**Conclusions:**

Identification of the transcription factors that mediate Pr77 transcription may help to elucidate how these retrotransposons are mobilized within the trypanosomatid genomes and their roles in gene regulation processes in this human parasite.

**Graphic abstract:**

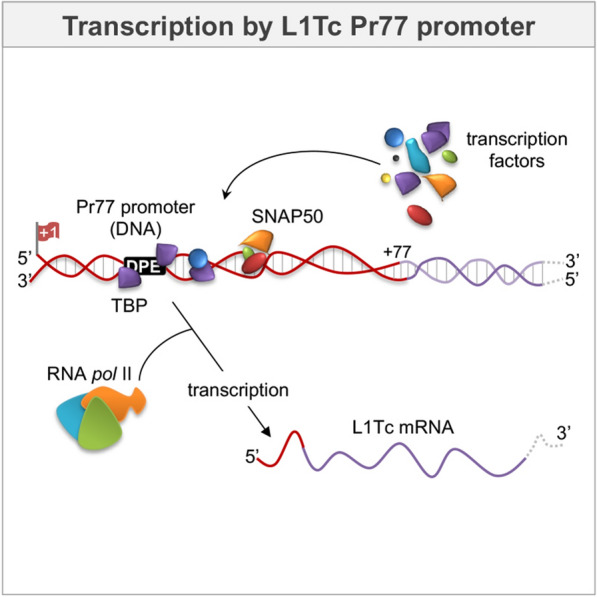

**Supplementary Information:**

The online version contains supplementary material available at 10.1186/s13071-021-04803-5.

## Background

Trypanosomatids are unicellular protozoa and aetiological agents of serious diseases affecting humans, such as Chagas disease (*Trypanosoma cruzi*), sleeping sickness (*Trypanosoma brucei*) and leishmaniasis (*Leishmania* spp.). Aside from the importance of these parasites to human health [1–3], the specific molecular properties that they exhibit, such as unusual genome organization, gene transcription and regulation [4], make them interesting study models. Trypanosomatid genomes organize their genes into large clusters (measuring over 100 kb in length) that are polycistronically transcribed by RNA polymerase II (RNAPII), with mRNAs maturing through spliced leader (SL) *trans*-splicing and polyadenylation-coupled processes. In addition, the genomes of these organisms are colonized by the non-LTR retrotransposons LINEs (long interspersed nuclear elements), such as L1Tc and *ingi* from *T. cruzi* and *T. brucei*, respectively; LINEs are autonomous retroelements that encode the enzymatic machinery that mediates their own transposition. Similarly, the non-LTR retrotransposons SINEs (short interspersed nuclear elements), such as NARTc and RIME from *T. cruzi* and *T. brucei*, respectively, which also colonize trypanosomatid genomes, are mobilized *in trans* by the LINE machinery, as they lack coding capacity. *ingi*/RIME and L1Tc/NARTc are the most abundant retroelements in the *T. brucei* and *T. cruzi* genomes and are not randomly distributed in the genome of *T. brucei* and *T. cruzi* as they are preceded by a conserved sequence, which may be the recognition site of the *ingi* and L1Tc-encoded endonuclease [5, 6]. In addition, trypanosomatid species contain degenerated retroelements, SIDERs (short interspersed degenerated retroposons) and DIREs (degenerate *ingi*/L1Tc-related elements), which have accumulated a large number of mutations that disable their mobilization capacity [7, 8].

L1Tc, the best-characterized LINE retrotransposon at the molecular and enzymatic levels, has a single ORF that encodes the enzymatic activities required for its mobilization [9–13]. L1Tc is actively transcribed along the three stages of the *T. cruzi* life cycle by RNA polymerase II (unpublished laboratory data and [14, 15]). All trypanosomatid retrotransposons belonging to the *ingi*/L1Tc clade (LINEs, SINEs, SIDERs and DIREs) exhibit a highly conserved 77-nt sequence at their 5′ ends, known as the Pr77-hallmark [8, 16]. Pr77 constitutes a dual system, as it has promoter (Pr77) and HDV-like ribozyme (L1TcRz) activities at the DNA and RNA levels, respectively [17]. Pr77 is a TATA-less promoter that contains a DPE motif (at positions + 25 to + 28 relative to the + 1 transcription start site), which is capable of activating the transcription of downstream genes by RNAPII, thereby generating abundant unspliced and translatable transcripts [14, 15]. The autocatalytic activity of L1TcRz cleaves upstream of the + 1 nucleotide of L1Tc and NARTc, enabling the release of retrotransposon intermediate RNA from cellular cotranscripts [18]. From a mobilization perspective, the presence of both functions (promoter and ribozyme) reinforces the autonomous character of the retrotransposons that carry the Pr77 sequence. Moreover, it has been suggested that the wide distribution of the Pr77-hallmark dual system within trypanosomatid genomes may be related to the gene regulation processes in these organisms [19].

Little is known about the transcription factors and molecular interactions that mediate the transcription process in trypanosomatids, as they have been researched primarily through biochemical studies focused on SL RNA gene transcription, particularly in *T. brucei*, *Leptomonas seymouri* and *Leishmania tarentolae*. To date, the SL RNA gene promoter and Pr77-hallmark are the only two RNAPII-dependent promoters reported in trypanosomatids. The SL RNA gene promoter provides the cap structure (m7GpppG) to the 5′ end of the *trans*-spliced matured mRNAs to be translated. SL RNA is a unique trypanosomatid RNA that is transcribed monocistronically and bears a TATA-less gene promoter that contains a bipartite proximal sequence element (PSE). Several general transcription factors that participate in SL RNA synthesis have been identified in *T. brucei*. These transcription factors include TATA-binding protein (TBP) also known as TBP-related factor 4 (TRF4) [20–22] and small nuclear RNA-activating protein complex (SNAPc) [23, 24] at an early stage for subsequent recruitment of other protein factors, such as TFIIA [23] and TFIIH [25–27], followed finally by recruitment to the RNAPII complex with TFIIB [28, 29].

It has been determined that TbTBP tightly associates in a specific way with SNAPc proteins and TFIIA, constituting a central core within the SL promoter, by recognizing specific sequences in this promoter [22–24]. Recruitment of trypanosomatid TBP to the SL RNA-gene promoter was also demonstrated for *Leishmania tarentolae* [30]. In this organism, TBP is part of a complex that includes three divergent components of the SNAP complex, at least one TFIIA orthologous subunit and a very divergent version of TFIIB [20, 22–24, 28, 29, 31].

SNAPc is formed by several proteins responsible for the transcription of small nuclear RNA genes (snRNAs) via RNAPII or RNAPIII, where the presence (RNAPIII) or absence (RNAPII) of the TATA sequence determines the type of polymerase that transcribes the gene. Trypanosomal SNAPc (tSNAPc) possesses a trimeric structure that contains SNAP50, SNAP42 and SNAP26 proteins. tSNAP50 is orthologous to the human SNAP50 subunit, which plays a critical role in the transcription of snRNA, while the other two subunits differ considerably from mammalian subunits. tSNAP26 appears to be a truncated version of human SNAP43, while tSNAP42 is unique to trypanosomes [32, 33].

In this paper, the proteins that specifically bind to the RNAPII-mediated Pr77 promoter sequence of trypanosomatid retrotransposons were analysed. Thus, it is reported that TBP and SNAP50 proteins interact with the Pr77 DNA promoter sequence and specifically bind to the double-stranded Pr77 sequence. TBP and SNAP50 have also been identified as components of the complex formed by the Pr77 sequence and nuclear proteins in *T. cruzi*. This report describes the existence of direct binding between rTBP and Pr77 sense and antisense sequences that is not affected by the structure of Pr77 and identifies the DPE motif-bearing sequence and the DPE downstream region as the preferential TBP docking regions in the Pr77 sequence. These results will help elucidate how the Pr77-mediated transcriptional process takes place in these parasites.

## Methods

### Parasite cultures, purification of genomic DNA and total, nuclear and cytoplasmic protein extracts

*T. cruzi* epimastigotes (Y and CL Brener strains) were grown at 28 °C in liver infusion tryptose (LIT) supplemented with 10% heat-inactivated fetal bovine serum (FBS, Flow Laboratory, Irvine, UK). Following lysis of parasites with 1% NP40 and nuclear lysis through the addition of 1% SDS, genomic DNA was purified by phenol–chloroform extraction and ethanol precipitation.

Purification of parasite proteins was performed in epimastigotes (Y strain) at the logarithmic phase of growth. Purification of the nuclear and cytoplasmic protein extracts was performed as detailed by Macias et al*.* [15]. Total protein extracts (TP) were obtained from parasites in the logarithmic growth phase, as described by Heras et al*.* [14]. Protein concentration was determined using the Micro BCA Protein Assay Kit (Thermo Scientific®), and quality was checked by SDS polyacrylamide gel electrophoresis (SDS-PAGE).

### Cloning and purification of the rTBP and rSNAP50 recombinant proteins

The TBP coding sequence (GenBank accession no. **AF465747.1**) was amplified by polymerase chain reaction (PCR) using *T. cruzi* genomic DNA (Y strain) as the template and TBP 5′ BamHI (5′GGTATGGATCCTTGGATGATGACTTTGACAAC3′) and TBP 3′ HindIII (5′GGTATAAGCTTTTACTTCTTTGCGTATTGTGC3′) oligonucleotides, which included the BamHI and HindIII restriction sites (underlined), respectively. The SNAP50 coding sequence (GenBank accession no. **XM_803702**) was PCR-amplified using the *T. cruzi* genomic DNA (CL Brener) strain as a template and 5′ BamH1TSNAPnoATG (ATAGGATCCCCGTCAAGAGGGGCGTTGGA-3′) and 3′ TSNAPHindIII (5′ATAAAGCTTTCACATTGTAAAATACTTCCC-3′) primers, which also included the BamHI and HindIII restriction sites (underlined). Following BamHI and HindIII digestion, the 0.8-kb (TBP) and 1.3-kb (SNAP50) amplified fragments were directly cloned into the pQE_30_ vector (Qiagen) digested with the same enzymes to generate pQE_30_TBPTc and pQE_30_SNAP50Tc clones. The sequence was confirmed by sequencing both DNA strands of the two constructs.

The TBP and SNAP50 recombinant proteins (rTBP and rSNAP50) were produced in the *Escherichia coli* M15 strain (Qiagen®) by the addition of 0.1 mM isopropyl β-d-thiogalactoside (IPTG) to the bacterial cultures overnight at 16 °C (rTBP) and for 3 h at 37 °C (rSNAP50). Proteins were extracted by the resuspension of bacterial pellets in either TBP buffer (20 mM Tris–HCl pH 7.9, 300 mM NaCl, 0.1% Triton X-100, 10% glycerol, 2 mM PMSF, 10 mM imidazole and 1 mg/ml lysozyme, pH 8) or SNAP50 buffer (50 mM phosphate buffer pH 8, 300 mM NaCl, 0.05% SDS, 1 mM PMSF and 1× protease inhibitor cocktail) and sonication for 8 min with pulses at 40%. rTBP and rSNAP50 were purified to homogeneity by Ni^+2^ affinity chromatography. Following incubation of each solubilized protein with Ni^+2^-nitrilotriacetic acid (NTA) resin (Qiagen) for 1 h at 4 °C, TBP resin was washed three times with TBP buffer containing 10, 20 and 30 mM imidazole and SNAP50 column twice with SNAP50 buffer at pH 8 and pH 7. The rTBP was eluted in the same buffer including 100 mM imidazole and rSNAP50 was eluted in the same solution at pH 6. Eluted fractions were resolved by SDS-PAGE and visualized through Coomassie blue staining. The protein concentration was determined by the Micro BCA™ Protein Assay Kit (Thermo Scientific).

### Hybridoma generation and production of αTBP and αSNAP50 monoclonal antibodies (mAbs)

Two BALB/c mice per group received two intraperitoneal inoculations (25 µg each) of both rTBP and rSNAP50 proteins and also received one intravenous dose (25 µg) at 2-week time intervals in any case. Anti-rTBP and anti-rSNAP50 specific antibodies (αTBP and αSNAP50) were detected in mouse sera by ELISA 2 weeks after the last immunization. Hybridomas were generated as described by Morón et al. [34]. Briefly, hybridomas were generated by fusion of splenocytes to murine myeloma SP2 cells by addition of PEG-400 and dispensed in 96-well culture plates containing RPMI-HAT medium supplemented with 20% heat-inactivated fetal bovine serum. Specificity and sensibility of the generated anti-TBP specific-antibodies were analysed by standard ELISA, western blot and indirect immunofluorescence. Cells from the ELISA-positive wells were cloned at least twice by limiting dilution. All animal protocols were approved by the Junta de Andalucía Public Health Animal Care Committee (Reference 29/11/2016/176) and by the IPBLN-CSIC Ethical Committee for Animal Procedures (CTC.01,05/16,CEEA). Antibody-producing hybridomas were grown and monoclonal antibodies (MoAbs) were purified from the cell culture supernatants by chromatography after passing the supernatants through a protein G-conjugated Sepharose (GE Healthcare) column. Columns were washed with 1× phosphate-buffered saline (Gibco) and MoAbs eluted in 0.1 M glycine pH 2.5. pH of eluted fractions was equilibrated by addition of 1 M Tris–HCl, pH 9.

### Protein identification by western blotting

Recombinant proteins (rTBP and rSNAP50) and total (TP), cytoplasmic (CP) and nuclear (NP) protein extracts from *T. cruzi* epimastigotes were boiled for 10 min at 95 °C and resolved in 14% or 10% SDS-PAGE as described by Sambrook et al. [35]. Gels were transferred to a PVDF membrane (Millipore) using the Mini-PROTEAN system (Bio-Rad). EMSAs were transferred to a PVDF membrane using a semidry transfer system (Biometra Fast-blot B33). Membranes were incubated in blocking solution (1× TBS containing 0.1% Tween-20) for 15 min and subsequently incubated with anti-6× His tag antibody at a 1:10000 dilution, anti-TcTBP antibody at a 1:1500 dilution or anti-TcSNAP50 antibody at a 1:5000 dilution overnight at 4 °C with gentle shaking. Anti-*T. cruzi* Histone H2A at a 1:500 dilution and anti-*Leishmania* cytoplasmic tryparedoxin peroxidase (cTXNPx—1:6000) antibodies were included as fractionation controls of nuclear and cytoplasmic fractions. Following washing with blocking solution, anti-mouse IgG peroxidase-linked antibody (Sigma) or anti-rabbit IgG peroxidase-linked antibody (Sigma) was employed at a 1:20000 dilution. The membranes were developed using the SuperSignal West Pico Chemiluminescent Substrate detection kit (Thermo Scientific) following the instructions of the manufacturer, and the results were visualized by exposure to CL-XPosure™ Film (Thermo Scientific).

### Protein capture assay

A total of 500 µg M-280 magnetic bead suspension (Dynabeads M-280 Streptavidin; Invitrogen®) was washed twice for 3 min with 100 µl of preparation buffer (1× PBS, 0.1% BSA). Beads were suspended twice in equilibration buffer (5 mM Tris–HCl, 0.5 mM EDTA, 1 M NaCl) and incubated for 3 min in a rotation wheel. Next, 80 pmol of each double-stranded DNA biotinylated probe (Pr77, SL and IL6 suspended in equilibration buffer) was added to the magnetic beads and incubated for 40 min at room temperature. Magnetic beads were equilibrated in 1× binding buffer (4 mM Tris–HCl pH 8, 12 mM HEPES, 1 mM EDTA, 0.5 mM DTT, 6% glycerol, 1 µg poly (dI-dC), 1 mM PMSF and 1× protease inhibitor cocktail) for 5 min and incubated with 1.5 mg of total extract proteins of the parasite in gentle shaking for 30 min at room temperature. Following washing of the dsDNA probe-bead-protein mixtures with 1× binding buffer, the proteins specifically attached to each DNA probe were eluted following incubation with 500 U of benzonase (Sigma®) in 30 µl of 1× elution buffer (PBS 1×, RQ1 DNase buffer 1× Promega®, 1 mM PMSF, and protease inhibitor cocktail 1×) for 30 min at room temperature. Each fraction, eluted (E), flow-through (FT) and beads without DNA (no DNA), employed as controls was resolved in 10% SDS-PAGE gels and subjected to western blot analysis.

### Indirect immunofluorescence protein co-localization assay

Epimastigotes in the logarithmic phase of growth were washed in cold 1× PBS, harvested by centrifugation at 2500 rpm for 5 min (4 °C), fixed by addition of 2% paraformaldehyde and attached to glass slides previously treated with 0.01% polylysine. A total of 6 × 10^4^ parasites/slide were permeabilized by incubation with PBS containing 0.1% Triton X-100, washed with PBS and incubated with blocking solution (1× PBS, 0.2% BSA) for 1 h in a wet chamber (Petri plate). Slides were subsequently incubated with anti-SNAP50 (1:100) and anti-TBP (1:100) antibodies for 1 h, washed with blocking solution and incubated with Alexa Fluor© 488 goat anti-mouse IgG (H+L) for SNAP50 and Alexa Fluor© 594 goat anti-mouse IgG (H+L) for TBP (Invitrogen®) at 1:2000 in blocking solution for 1 h. Since both monoclonal antibodies (αSNAP50 and αTBP) were generated in mice, a blocking step using blocking Fab’ antibodies (Sigma®) at a 1:1000 dilution for 1 h was performed before the addition of the anti-TBP antibody. Parasites were DAPI stained with Prolong Gold Antifade Mountant (Invitrogen®) and visualized in a Zeiss Axio Imager A1 fluorescence microscope. Images were acquired and analysed using ImageJ MBP software (Mac Biophotonics®).

### Generation of dsDNA probes and competitors for EMSA (electrophoretic mobility-shift assay) and EMSA competition

All oligonucleotides used in this work (see Additional file 3: Table S3 for details) were purified before use by electrophoresis through denaturing polyacrylamide gels, as described previously [11]. One oligonucleotide of each pair (or single strand, if applicable) was 5′ end-labelled using ATP^32^ (Perkin Elmer®) and T4 polynucleotide kinase (Roche®), and unincorporated isotopes were removed by gel filtration chromatography (Sephadex G25). Double-stranded DNA probes (dsPr77, ds1-24, ds12-33, ds24-51, ds29-51, ds52-77, dsM1 and dsM2) were generated by heating each ^32^P 5′ end-labelled oligonucleotide with its reverse complementary oligo at 95 °C for 5 min and cooling the mixture to room temperature (22 °C), as described by Heras et al. [11].

The dsPr77 DNA competitor was obtained by hybridization of Pr77s and Pr77as unlabelled oligonucleotides. The dsAPT DNA pool competitor (measuring 77 bp in length) was generated by PCR relative to the third round of molecular selection-enrichment by Systematic Evolution of Ligands by Exponential Enrichment (SELEX) and Poly (dI-dC) nonspecific DNA competitor obtained by Sigma®. Oligonucleotide names and sequences are presented in Additional file 3: Table S3.

For EMSA, 0.33 nM ^32^P-dsPr77 was incubated with increasing amounts of rTBP or rSNAP50 (0.32–6.35 μM) in 20 μl of binding buffer 1 (20 mM Tris pH 7.9, 60 mM KCl, 5 mM MgCl_2_, 10 mM DTT, 6% glycerol and 0.2 mg/ml BSA) for 30 min at 37 °C. Reactions were stopped by the addition of 8 μl of dye solution (50% glycerol, 0.1% bromophenol blue and 0.1% xylene cyanol). Nucleic acid-protein complexes were resolved on 6% native polyacrylamide gels containing 1% glycerol and 4 mM MgCl_2_ in 1% Tris–borate-EDTA buffer. For competition experiments, 0.125 nM ^32^P-dsPr77 was mixed with 1.58 μM rTBP and increasing amounts (5-, 10-, 50-, 100-, 250- and 500-fold excess) of cold dsPr77 or aptamer dsAPT or poly(dI-dC) competitors and incubated at 37 °C for 30 min. Each radio-labelled probe was included in the corresponding assay and referred to as a free probe (FP). EMSA under denaturing conditions was performed by the addition of 1, 3, 5 and 10% dimethyl sulfoxide (DMSO) to the rTBP-^32^P-dsPr77 binding reaction. Denaturing of oligos corresponding to the sense (s) and antisense (as) sequences of Pr77 was performed at 95 °C for 5 min, and probes were subsequently incubated with rTBP for 15 min at 4 °C to prevent renaturing.

EMSA using the preformed ^32^P-labelled oligo pairs was performed with 0.33 nM of each oligo pair (1–24, 12–33, 24–51, 29–51, 52–77, M1 and M2) and 6.35 μM rTBP. The same amount of each oligo pair (100000 cpm) was incubated with 3 μg of nuclear protein extract in binding buffer 2 (12 mM HEPES, 4 mM Tris–HCl pH 8, 1 mM EDTA pH 8, 125 ng/μl BSA, 2 μg poly (dI-dC), 0.5 mM DTT and 6% glycerol) for 30 min at room temperature. Competition assays (Additional file 2: Figure S2) were performed by incubation of rTBP protein (1.58 μM) with ^32^P-dsPr77 (0.125 nM) probe and increasing amounts (5-, 10-, 50- and 100-fold excess) of each oligo pair in binding buffer 1 at 37 °C for 30 min. Complexes formed between ^32^P-dsPr77 (0.33 nM) and 6.35 µM of the recombinant proteins (TBP, SNAP50, BSA, KMP11 or T-GP63) were generated in binding buffer 1, as described previously for EMSA, and crosslinked by UV irradiation (254 nm; 3000 W/cm^2^) in a CL-1000 ultraviolet crosslinker (UVP®) for 10 min. All EMSA gels were dried, and complexes and free probes (FP) were visualized by PhosphorImager analysis. Products were quantified using ImageQuant 5.2 (Amersham Biosciences) software.

### EMSA crosslinking for TBP and SNAP50 immunodetection

For the detection of TBP and SNAP50 in dsPr77-parasite protein complexes of reduced motility, 100000 cpm of ^32^P-dsPr77 and 8 pmol (~ 400 ng) of cold dsPr77 were incubated with 3 and 60 µg of nuclear or 180 µg of total protein extracts in binding buffer 2, as described above, and reactions were subjected to UV radiation as indicated above. Reactions were denatured by heating and resolved by 10% acrylamide SDS-PAGE or 6% native acrylamide gel electrophoresis. Gels were transferred to PVDF membranes through semidry transfer (Biometra Fast-blot B33) and analysed by western blotting using anti-TBP (1:150) and SNAP50 (1:5000) antibodies.

## Results

### *T. cruzi* TBP and SNAP50 proteins bind to the Pr77 promoter sequence

**Fig. 1:**
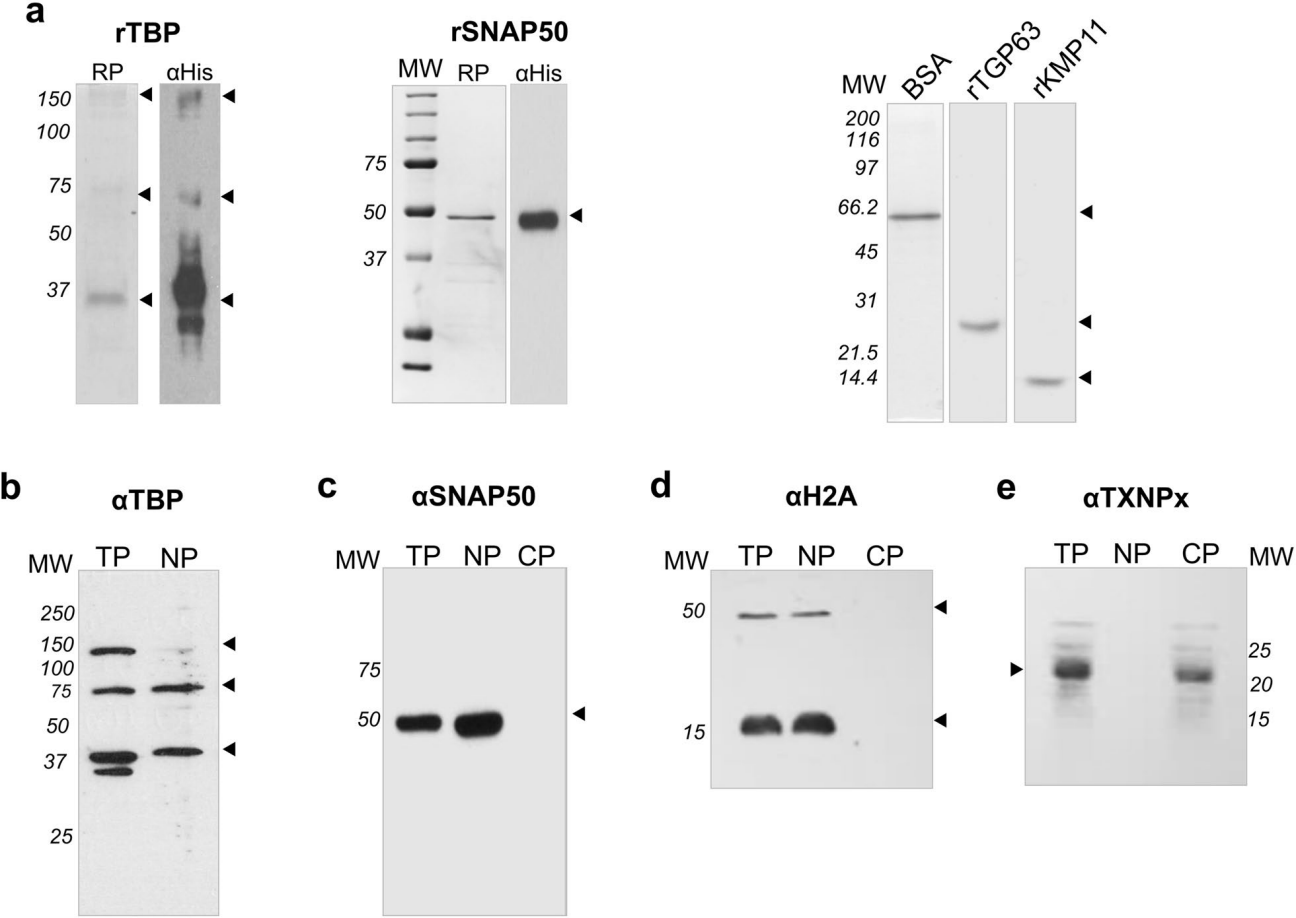
Purification of recombinant proteins and detection of TBP and SNAP50 in *T. cruzi* protein extracts. **a** Purification of *T. cruzi* rTBP, rSNAP50, rTGP63 and rKMP11 recombinant proteins. All proteins were purified to homogeneity by Ni^+2^ affinity chromatography, electrophoresed on a 10 and 14% SDS-PAGE and visualized following Coomassie blue staining (RP in rTBP, RP in rSNAP50, rTGP63 and rKMP11). Bovine serum albumin was employed as control (BSA). rTBP and rSNAP50 proteins were also analysed by western blotting using anti-6×His antibody (lanes αHis in rTBP and rSNAP50). Detection of **b** TBP, **c** SNAP50, **d** Histone H2A and **e** cytoplasmic tryparedoxin peroxidase (cTXNPx) in *T. cruzi* total (TP), nuclear (NP) and cytoplasmic (CP) protein extracts by specific MoAbs generated against rTBP and rSNAP50 proteins and polyclonal antibodies produced against *T. cruzi* Histone H2A (in rabbit) and *Leishmania* tryparedoxin peroxidase (cTXNPx, in mouse) by western blotting. *MW* molecular weight marker in kDa

To investigate whether the TBP and SNAP50 proteins interact with the Pr77 DNA promoter sequence, an immunoassay was performed that included capture of parasite proteins that specifically bind to Pr77 double-stranded DNA (dsDNA) and subsequent protein identification by western blotting. To this end, the recombinant TBP (~ 37 kDa) and SNAP50 (~ 50 kDa) proteins (Fig. 1a, RP in panels rTBP and rSNAP50, respectively) as well as specific monoclonal antibodies (MoAbs) against them were generated. Homologous fractions of rTBP and rSNAP50 were transferred to PDVF membranes and analysed by western blotting employing the anti-6×-His antibody. As observed in Fig. 1a, under the experimental conditions employed the anti-6× His tag antibody recognized three bands of 37, 75 and 150 kDa in the rTBP lane (Fig. 1a, lane rTBP αHis), which are consistent with the monomeric, dimeric and tetrameric forms of the rTBP, and a band of 50 kDa in the lane of rSNAP50 (Fig. 1a, lane rSNAP50 αHis), which is consistent with the monomeric form of the SNAP50 protein. The kinetoplast membrane protein of 11 kDa (KMP11) and a fragment of major surface glycoprotein (rT-GP63) recombinant proteins were also purified to be utilized as controls in the subsequent experiments (Fig. 1a, lanes rKMP11 and rT-GP63). Bovine serum albumin (BSA) was also used as a control (Fig. 1a, lane BSA).

The specificity of the MoAb was tested against total (TP), cytoplasmic (CP) and nuclear (NP) soluble protein extracts from *T. cruzi* epimastigote forms by western blot analysis. TBP hybridoma against TBP was selected for further investigation because it recognized proteins of approximately 37, 75 and 150 kDa in the epimastigote total and nuclear soluble extracts and although at different proportions (the 150 kDa fraction was less abundant in the nuclear soluble extracts) (Fig. 1b, lanes TP and NP), the detected proteins had the same molecular weight as those detected in the purified rTBP fraction by this MoAb (data not shown) and by the αHis (Fig. 1a, lane rTBP αHis), which was in keeping with the monomeric, dimeric and tetrameric status of the protein. Monoclonal antibodies produced by the SNAP50-hybridoma specifically recognized a single protein of approximately 50 kDa in *T. cruzi* total protein extract (Fig. 1c, TP) and nuclear protein extract (Fig. 1c, NP), whereas it did not detect any proteins in the cytoplasmic fraction (Fig. 1c, lane CP). Specific antibodies raised against *T. cruzi* Histone H2A [36] and *Leishmania* cytoplasmic tryparedoxin peroxidase (cTXNPx) [37] were employed as controls to verify the proper fractionation between nuclei and cytoplasm (Fig. 1d, e, respectively).

**Fig. 2:**
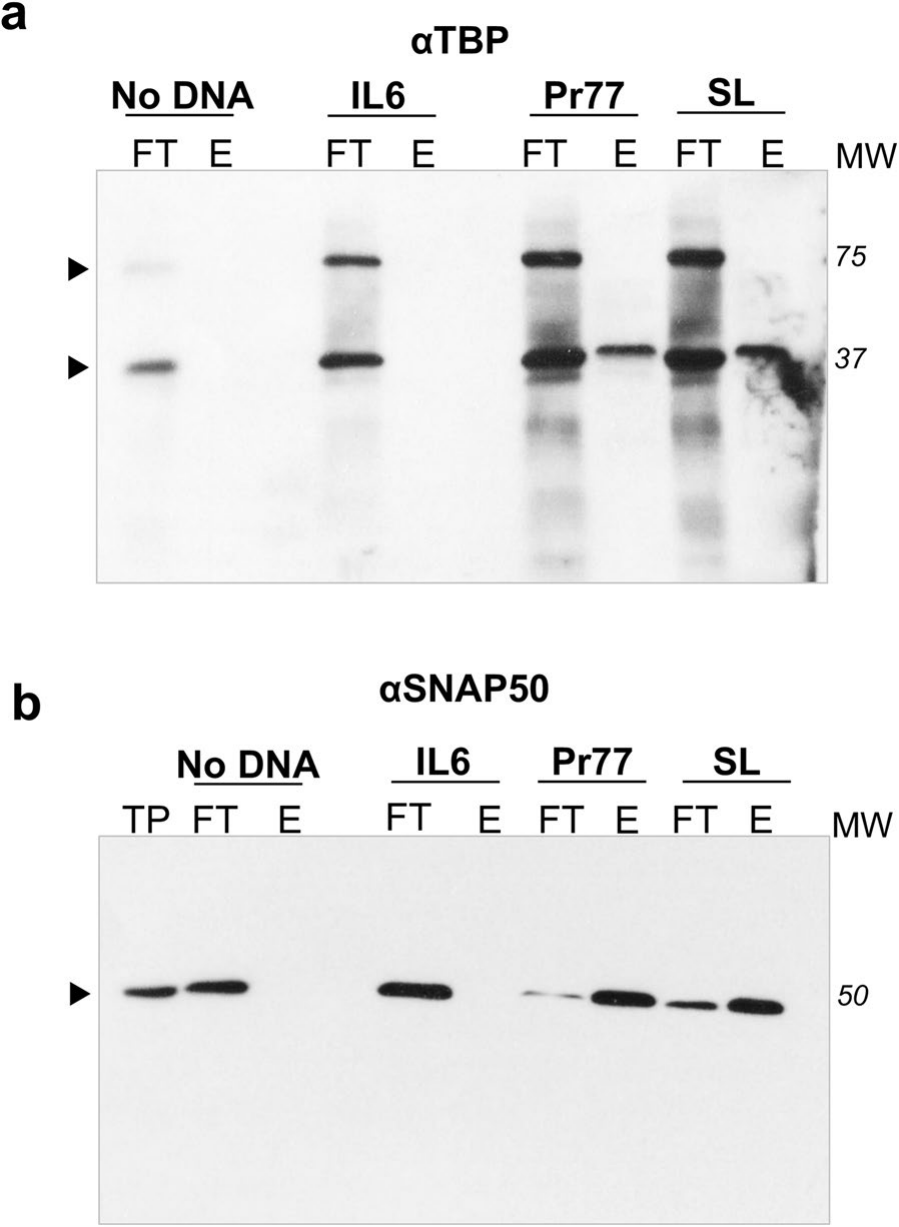
Identification of *T. cruzi* TBP and SNAP50 proteins in the protein complex specifically bound to the Pr77 sequence. **a** Dynabeads protein capture assay coupled to western blotting was performed by incubating 1.5 mg of *T. cruzi* total protein extract with 80 pmol of the biotinylated double-stranded DNA probes L1TcPr77, *T. cruzi* SL and human IL6. Beads without a DNA probe (No DNA) were used as a negative control. Flow-through (FT) and eluted (E) fractions following benzonase treatment were loaded into a 10% SDS-polyacrylamide gel and transferred to nylon membranes for western blotting analyses employing **a** anti-TBP and **b** anti-SNAP50 specific antibodies. Ten micrograms of total protein extract (TP) was used as a positive control. Detected bands are labelled by a black arrowhead. Molecular weight marker (MW) is shown in kDa

To capture the parasite proteins that specifically bind to the Pr77 promoter sequence, a biotinylated dsPr77 DNA probe was coupled to streptavidin-coated magnetic Dynabeads and subsequently incubated with soluble total proteins of *T. cruzi* epimastigotes. The unbound proteins (flow-through fraction, FT) and eluted proteins (E) were resolved by 10% PAGE and subsequently analysed by western blot analysis using anti-TBP- or anti-SNAP50-specific MoAbs. The 80-bp biotinylated SL-dsDNA corresponding to the spliced leader (SL) gene promoter sequence [38] and 78 bp IL6-dsDNA corresponding to the human interleukin 6 (IL6) gene were employed as positive and negative binding controls, respectively. In addition, Dynabeads without DNA probes were also assayed as a negative control (No DNA). The obtained results indicate (Fig. 2a, b) that both TBP and SNAP50 specifically bind to the Pr77 promoter, since protein bands of 37 and 50 kDa, respectively, are recognized in the elution fractions (lanes Pr77-E in Fig. 2a, b). In the case of TBP, the eluted fraction contained the monomeric form of the protein (Fig. 2a, lane Pr77-E), although the anti-TBP antibody was also capable of detecting the multimeric forms of the TBP protein in the flow-through (FT) unbound fractions (Fig. 2a, lanes FT in No DNA, IL6, Pr77 and SL), as seen in TP and NP extracts of the parasite (Fig. 1b, lanes TP and NP). In addition, TBP and SNAP50 proteins were also shown to specifically bind to the SL-dsDNA probe, as the same single hybridization bands of 37 and 50 kDa were also detected in the eluted fraction that was incubated with the anti-TBP- and anti-SNAP50-specific MoAbs, respectively (lanes SL-E of Fig. 2a, b, respectively). In contrast, the TBP and SNAP50 parasite proteins were not immunodetected when the IL6-dsDNA probe or magnetic beads without DNA were tested (lanes IL6-E and No DNA-E in Fig. 2a, b, respectively). As expected, the anti-TBP-specific antibody recognized the monomeric (37 kDa) and dimeric (75 kDa) forms of the TBP protein in the total extracts of the parasite (lanes Pr77-FT, No DNA-FT, IL6-FT, SL-FT of Fig. 2a), and the SNAP50-specific antibody detected a monomeric form of SNAP50 in the whole lysates of the parasite (lanes Pr77-FT, No DNA-FT, IL6-FT, SL-FT in Fig. 2b). Taken together, these results indicate that the monomeric forms of TBP protein specifically binds to the Pr77 promoter sequence.

### *T. cruzi* TBP and SNAP50 colocalize in the parasite nucleus

**Fig. 3:**
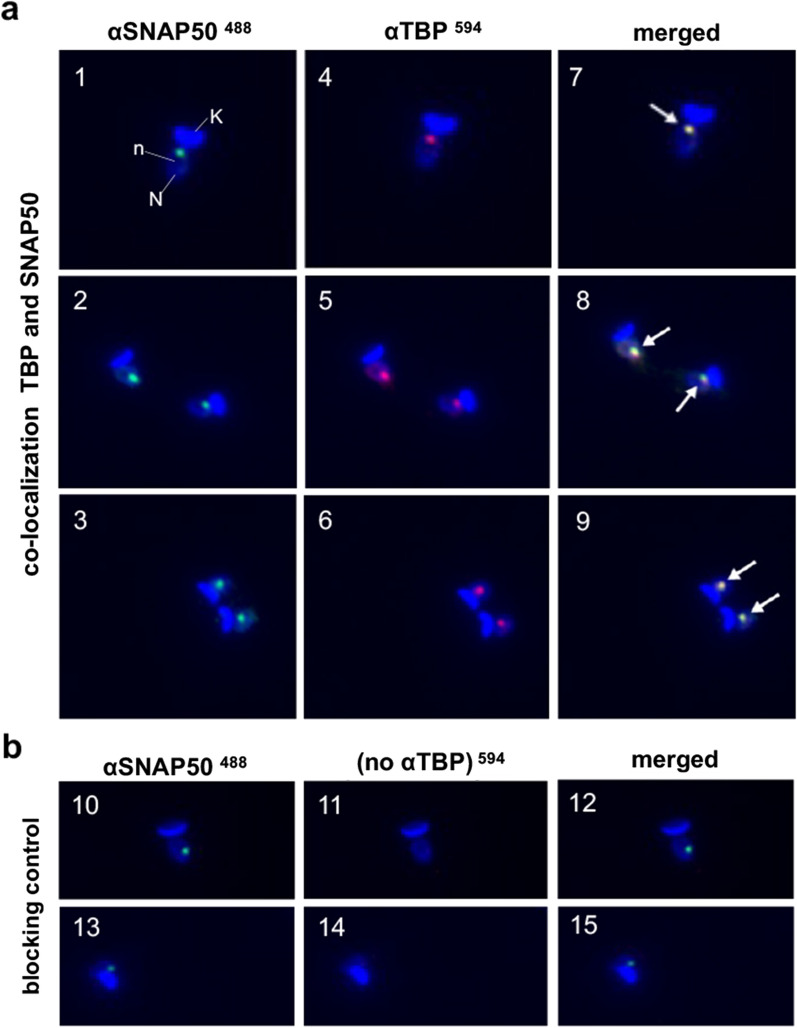
Colocalization of the TBP and SNAP50 proteins in the *T. cruzi* nucleus by immunofluorescence microscopy. **a** Immunodetection of TBP and SNAP50. Indirect immunofluorescence was performed by fixing 6 × 10^4^ permeabilized epimastigotes per slide and incubation with a specific antibody against SNAP50 (dil. 1:100) and Alexa Fluor© 488 goat anti-mouse IgG (H+L) (dil. 1:2000) as secondary antibody (panels 1, 2 and 3). Following incubation with anti-Fab’ fragment antibody (dil. 1:1000), the slides were incubated with anti-TBP antibody (dil. 1:100) and subsequently with Alexa Fluor© 594 goat anti-mouse IgG (H+L) (dil. 1:2000) (panels 4, 5 and 6). Merged images of panels 1 and 4, 2 and 5 and 3 and 6 are shown as panels 7, 8 and 9, respectively. **b** Immunofluorescence carried out without anti-TBP antibody (blocking control). Homologous slides were incubated with anti-SNAP50 antibody, Alexa Fluor© 488 goat anti-mouse IgG (H+L) and anti-Fab’ fragment antibody (panels 10 and 13) followed by incubation with Alexa Fluor© 594 goat anti-mouse IgG (H+L) (panels 11 and 14). Panels 10–11 and 13–14 as merged images (panels 12 and 15). DAPI staining in **a** and **b** for visualization of parasite nuclei (N), nucleoli (n) and kinetoplasts (K). White arrows indicate colocalization of TBP and SNAP50 proteins

To determine the subcellular location of the TBP and SNAP50 proteins in *T. cruzi*, indirect immunofluorescence experiments were performed. For simultaneous detection and analysis of the localization of TBP and SNAP50 proteins into the parasite, epimastigote forms were first incubated with the monoclonal antibody directed against SNAP50 followed by incubation with the MoAb directed against TBP, as described in “Methods”. The obtained results shown in Fig. 3 indicate that SNAP50 is located at the parasite nucleus (Fig. 3a, images 1, 2 and 3). TBP-specific antibody also localized the corresponding TBP protein to the epimastigote nucleus (Fig. 3a, images 4, 5, 6). Merged images enabled to observe that SNAP50 and TBP proteins occupy a specific region in the parasite nuclei (Fig. 3, boxes 7, 8 and 9). As expected, the reaction performed in the absence of anti-TBP MoAb enabled the detection of SNAP50 (Fig. 3b, image 11) but not the observation of the previously detected colocalization pattern of SNAP50 and TBP proteins (Fig. 3b, merged image 12).

### TBP binds directly to the Pr77 promoter in a cooperative form, and SNAP50 exhibits weak direct binding to the Pr77 sequence

**Fig. 4:**
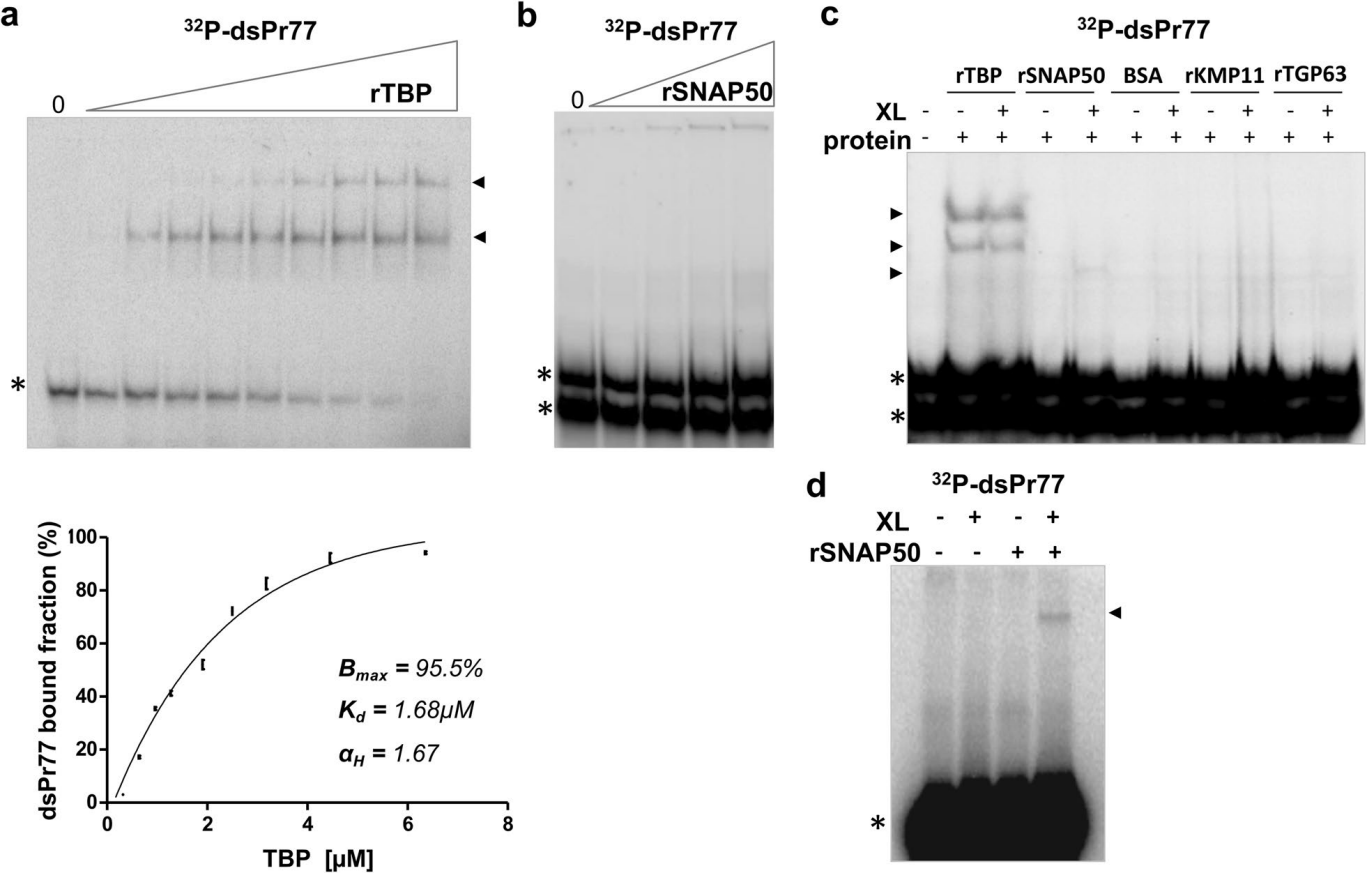
Binding analyses and binding kinetics of TBP and SNAP50 to the Pr77 sequence by EMSA. Increasing amounts (0.32–6.35 µM) of **a** (top panel) rTBP and **b** rSNAP50 proteins were incubated with 0.33 nM ^32^P-radiolabelled double-stranded Pr77 sequence (^32^P-dsPr77) as a probe. **a** Bottom panel: binding kinetics of rTBP to ^32^P-dsPr77. Binding data from three independent experiments, as shown in Fig. 4a and Figure S1, were fitted to the Hill model, and thermodynamic parameters *B*_max_ (maximum percentage of bound DNA), *K*_d_ (dissociation constant) and *α*_H_ (Hill coefficient) were calculated. **c** ^32^P-dsPr77 (0.33 nM) was incubated with 6.35 μM TBP, SNAP50, BSA, KMP11 and T-GP63 proteins (+), and binding reactions (+) were subjected to UV-crosslinking (XL). **d** ^32^P-dsPr77 (0.33 nM) was incubated with 6.35 μM rSNAP50 protein, and binding reactions were subjected (+) to UV-crosslinking (XL). Reactions performed with the same amount of P^32^-dsPr77 and without protein were subjected to UV-crosslinking (+) as a control. After incubation at 37 °C for 30 min, reactions were resolved on 6% native polyacrylamide gels. The shifted complexes formed are indicated with a black arrowhead and free ^32^P-dsPr77 probe with an asterisk

To investigate the binding specificity of rTBP and rSNAP50 to the Pr77 DNA promoter sequence, EMSAs were performed using the ^32^P-radiolabelled double-stranded Pr77 sequence (^32^P-dsPr77) as a probe and increasing amounts of TBP or SNAP50 purified recombinant proteins (Fig. 4a, b, respectively). As controls of binding specificity to ^32^P-dsPr77, the purified rKMP11 and rT-GP63 recombinant proteins were also examined as well as BSA (see below). As observed in Fig. 4a, rTBP bound to the dsPr77 sequence, as a series of migrating bands of reduced mobility corresponding to Pr77-TBP binding complexes appeared (Fig. 4a black arrowheads), and their abundance increased as the protein concentration increased and the amount of Pr77 free probe (^32^P-dsPr77) decreased. This result supported the direct specific binding of TBP to dsPr77 DNA. A similar EMSA performed with increasing amounts of rSNAP50 and ^32^P-dsPr77 showed no formation of any complex of reduced mobility (Fig. 4b), suggesting that there is no direct binding of SNAP50 to the Pr77 sequence (Fig. 4b).

In addition, homologous fractions of each protein-Pr77 binding reaction were UV-crosslinked to stabilize the preformed protein-DNA complexes. Crosslinking of the rTBP-dsPr77 reaction did not show any difference in the previously observed binding, as the same bands of reduced mobility were observed (Fig. 4c, lanes rTBP, XL− and +). However, crosslinking of the dsPr77-rSNAP50 reaction enabled us to observe a slight band of reduced mobility, which could be a consequence of specific but weak direct binding of rSNAP50 to dsPr77 (Fig. 4c, lane SNAP50 XL). Neither BSA nor the parasite proteins KMP11 and T-GP63, which were employed as protein-binding controls, exhibited any type of binding affinity to Pr77, even after UV-crosslinking (Fig. 4c, lanes BSA, rKMP11 and rT-GP63 XL− and +). The observed complex of reduced motility observed only when the rSNAP50 and dsPr77 DNA reaction was crosslinked appears to be a consequence of the UV-crosslinking-induced stabilization of a complex formed as result of binding of rSNAP50 to dsPr77 sequence (Fig. 4d, SNAP50+, XL+) and not due to a particular conformation of dsPr77 probe, since this complex was not observed in crosslinked reaction containing dsPr77 probe alone (Fig. 4d, lane SNAP50−, dsPr77+).

To evaluate the thermodynamic parameters of rTBP-dsPr77 binding complex formation, the data from three independent experiments (Additional file 1: Figure S1), performed as shown in Fig. 4a (top panel), were fitted to the Hill model. The protein concentration at which half of the dsPr77 probe remained bound to the protein (*K*_d_) was estimated to be 1.68 μM, indicating that TBP binds to the Pr77 sequence with high affinity (Fig. 4a, bottom panel). In addition, determination of the Hill coefficient (*α*_H_) indicated that TBP follows a cooperative binding model, as the Hill coefficient (*α*_H_) had a value of 1.67.

### TBP specifically binds along the Pr77 DNA promoter sequence

**Fig. 5:**
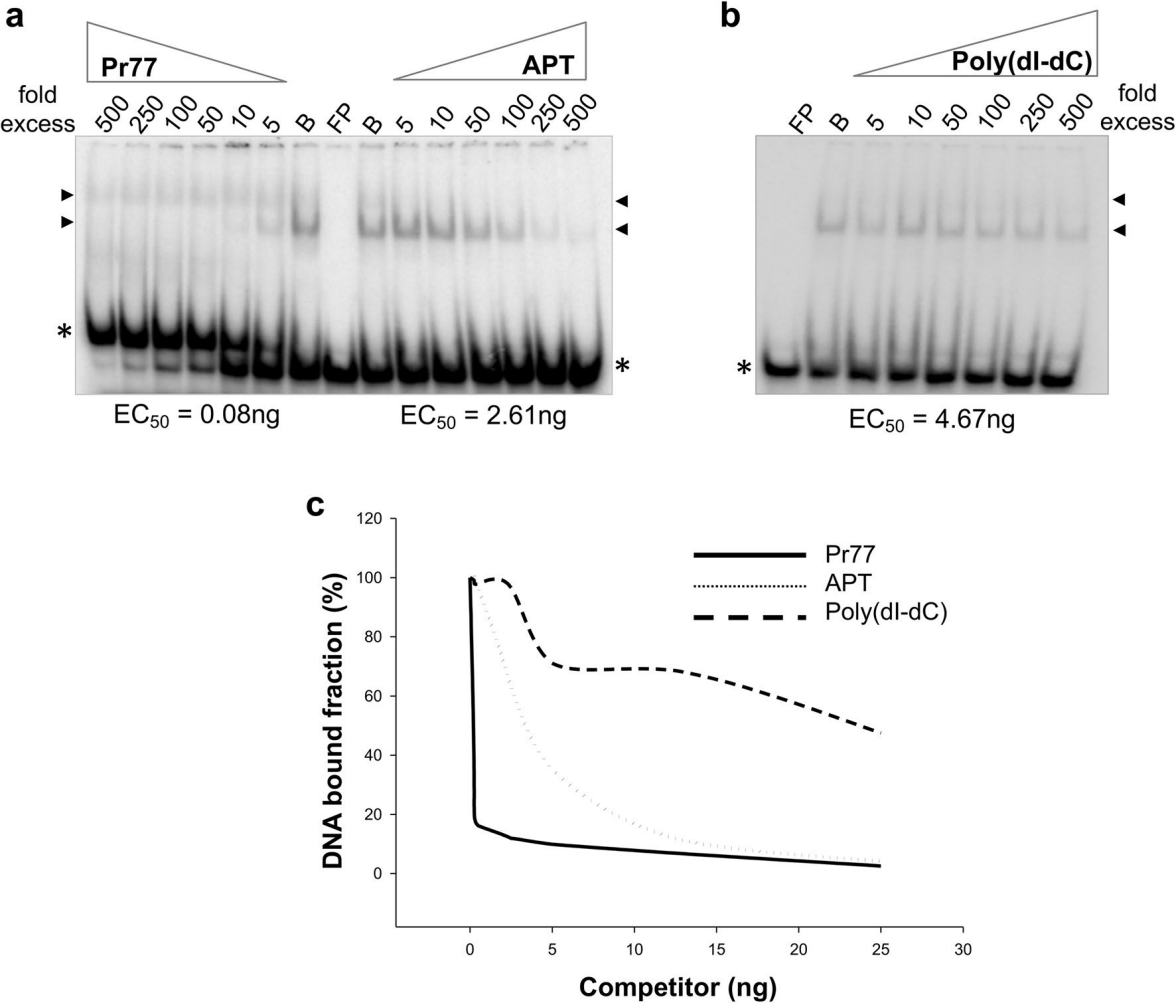
Binding specificity of TBP to the Pr77 sequence by EMSA competition. EMSA was carried out after preincubation of 0.125 nM ^32^P-dsPr77 with 1.58 μM rTBP and with different concentrations (as indicated, fold excess ranging from 5 to 500 times) of unlabelled DNA competitors: **a** dsPr77 sequence and APT (aptamer mixture) and **b** poly(dI-dC) (commercial dI-dC polymer). As a negative control, reactions were carried out without protein (FP lane, Free Probe) or without competitor (B lane, Binding reaction). Reactions were performed at 37 °C for 30 min and resolved in 6% polyacrylamide native gels. In **a** and **b**, the asterisks indicate the free form that ^32^P-dsPr77 adopts, and the black arrowhead indicates the formed complexes of higher molecular weight, which decrease in amount as the competitor concentration increases. **c** Fitting the competition kinetics data to a four-parameter logistic model with each competitor. Quantification was performed using ImageQuant software (Pharmacia)

To further investigate the binding specificity of the TBP protein to the Pr77 promoter sequence, a variety of DNA fragments were examined by EMSA competition experiments. Accordingly, a constant amount of ^32^P-radiolabelled double-stranded fragments corresponding to the Pr77 full-length sequence (^32^P-dsPr77) was incubated with increasing amounts of each double-stranded DNA unlabelled competitor. The reaction was performed at a protein concentration of rTBP that maintained some of the DNA probe at its free state and an amount of the ^32^P-dsPr77 probe forming part of the DNA–protein complexes. Unlabelled double-stranded Pr77 full-length sequence (Pr77), a pool of double-stranded 77 bp-long aptamers of randomly variable sequences (APT) and a nonspecific synthetic copolymer (poly dI-dC) were employed as DNA competitors. The data obtained in this experiment showed that the two shifted bands observed as a result of dsPr77 and rTBP complex formation (Fig. 5a, indicated by black arrowheads) were quickly displaced when cold dsPr77 was added to the reaction, since the addition of a fivefold cold dsPr77 probe displaced > 50% of the ^32^P-dsPr77-rTBP binding complex (Fig. 5a, left panel). The same amount of the APT and poly(dI-dC) competitors did not reach this degree of ^32^P-dsPr77-TBP complex displacement, since > 100- and at least 500-fold concentrations of APT and poly(dI-dC) competitors, respectively, were necessary to produce the same degree of displacement (Fig. 5a, APT and 5b). The effective concentration 50 (EC50), defined as the competitor concentration required to release half the amount of the protein bound to the radiolabelled DNA probe, was calculated by quantification of the shifted bands and fitting the experimental data to a four-parameter logistic curve (see Materials and Methods for details). The obtained results show EC50 Pr77 = 0.08 ng, EC50 APT = 2.61 ng and EC50 poly (dI-dC) = 4.67 ng. Neither the APT nor poly(dI-dC) competitors were capable of achieving complete displacement of the ^32^P-dsPr77-TBP complex (Fig. 5c). These findings suggest that TBP specifically binds to Pr77.

The influence of the Pr77 dsDNA secondary structure on the binding specificity of rTBP to this highly structured DNA was investigated next [11]. For this purpose, EMSA resolved in native gel was carried out using rTBP and dimethyl sulfoxide (DMSO) as denaturing agent of ^32^P-dsPr77.

**Fig. 6:**
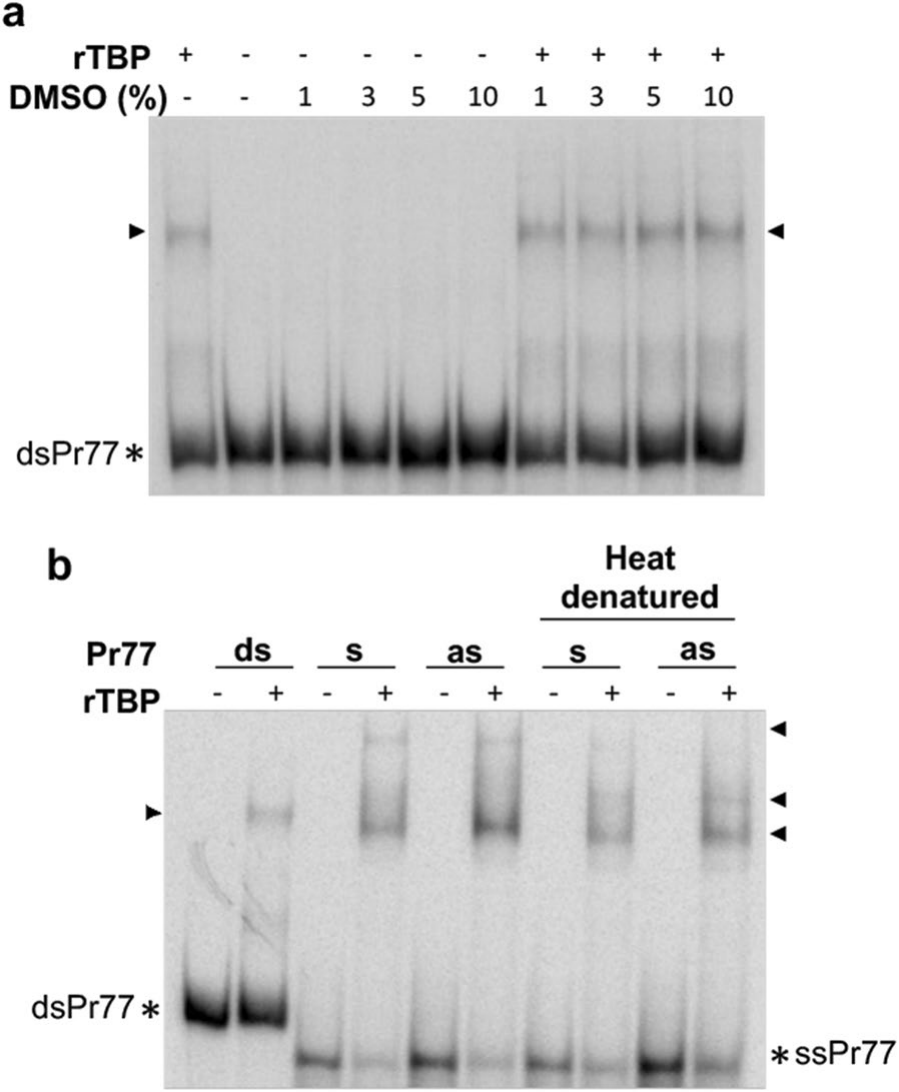
Influence of the Pr77 secondary structure on the binding capacity of rTBP to double-stranded and single-stranded Pr77 sequences. EMSA measuring the binding capacity of rTBP to **a** native and DMSO-denatured ^32^P-dsPr77 and to **b** native and heat-denatured sense- and antisense-single strands of the Pr77 sequence. **a** rTBP (6.35 μM) was incubated with 0.33 nM ^32^P-dsPr77 in the presence of DMSO at 1, 3, 5 and 10% (v/v) at 37 °C for 30 min. Protein-DNA binding reactions without DMSO (−) and reactions carried out with ^32^P-dsPr77 incubated with the same amounts of DMSO were included as controls. **b** rTBP (6.35 μM) was incubated with ^32^P-labelled native or heat-denatured (95 °C for 5 min) sense (s) and antisense (as) DNA strands corresponding to the Pr77 sequence for 15 min at 4 °C. The reaction carried out with ^32^P-dsPr77 (ds) was also included. All reactions in **a** and **b** were also performed in the absence of rTBP. Reactions were resolved in 6% polyacrylamide native gels. The black arrowhead indicates the formed complexes of reduced mobility, and the asterisks indicate the electrophoretic mobility of the nucleic acid-free conformations (dsPr77 and ssPr77)

As observed in Fig. 6a, TBP conserves its binding affinity to dsPr77 even when Pr77 is denatured with 10% DMSO, as the same complexes of reduced mobility were observed both with and without DMSO (Fig. 6a). Furthermore, the binding capacity of TBP for the single-stranded Pr77 sequence was also analysed by EMSA employing ^32^P-labelled 77-bp-long oligos corresponding to the sense and antisense single strands of the Pr77 sequence (Fig. 6b, s and as, respectively). At the same time, the participation of the secondary structure of these Pr77-derived single-stranded DNAs was studied by utilizing them as heat-denatured DNAs in the EMSA (Fig. 6b, heat-denatured sense and antisense, respectively). Thus, the single-stranded probes were heat-denatured and incubated with rTBP at 4 °C to prevent probe renaturing. dsPr77 DNA was included as a positive control of TBP binding to DNA. All reactions were also carried out without rTBP as control of the electrophoretic motility in native gel of each free DNA probe.

The obtained results show shifted bands as a result of complex formation between TBP and the sense and antisense strands of Pr77 independently of whether these sequences are endowed with their natural secondary structure or denatured (Fig. 6b), which indicates that rTBP is able to bind to both sense and antisense single-stranded Pr77 sequences in addition to dsPr77 and independent of the Pr77 conformation. Taken together, these data suggest that the specificity of TBP binding to Pr77 sequence is related to the nucleotide composition of Pr77 and not to the conformation that this sequence adopts.

### TBP and nuclear proteins from *T. cruzi* exhibit preferential binding sites along the Pr77 sequence

**Fig. 7:**
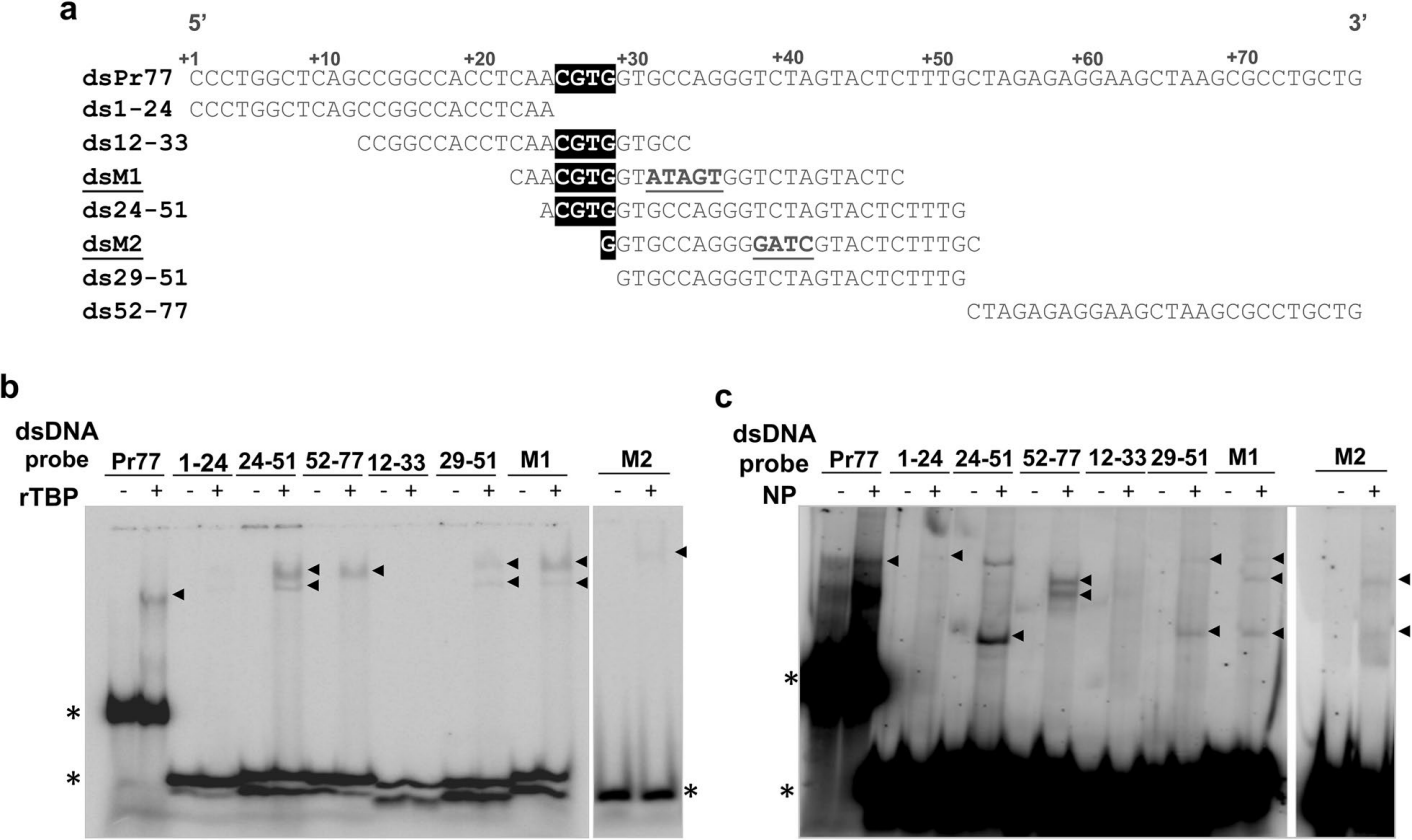
Mapping the preferential binding sites of rTBP and nuclear proteins to the Pr77 sequence. **a** Sequence of Pr77 and of the oligo pairs employed as probes for binding assays of **b** rTBP and **c** nuclear proteins (NP) of the parasite. In **a**, the DPE motif is shaded, and introduced point mutations are underlined. A total of 6.35 μM **b** rTBP or 3 μg of **c** NP was incubated with 0.33 nM or 100,000 cpm of each dsDNA probe in **b** and **c**, respectively at 37 °C for 30 min. All reactions were also carried out in the absence of **b** rTBP or **c** NP. Reactions were resolved in 6% polyacrylamide native gels. The black arrowhead indicates the formed complexes of reduced mobility, and the asterisks indicate the electrophoretic mobility of the nucleic acid-free conformations

To explore the binding ability of TBP in depth and to determine the TBP binding sites to the Pr77 sequence, EMSAs were performed using increasing amounts of rTBP and several overlapping ^32^P-radiolabelled DNA oligonucleotide pairs that mapped along the entire Pr77 sequence (Fig. 7a) and resolved in native gel. In addition, two oligo pairs containing point mutations that had previously been shown to abolish the Pr77 transcription capacity (M1 and M2) [15] were included in the assay (Fig. 7a).

The obtained results showed bands of reduced motility in most EMSAs which correspond to DNA–protein complex formation as a result of binding between rTBP and the oligonucleotide pairs (Fig. 7b). In the case of oligo ds12-33, no complex formation was observed at the protein and ^32^P-radiolabelled oligo concentrations that were analysed, even though this oligo mapped to the DPE motif and its upstream sequence. The strongest binding was observed between rTBP and oligo ds24-51, which contains the DPE and the DPE-downstream region, suggesting that TBP binds preferentially to DPE and its downstream sequence. The presence of complexes of reduced mobility in the EMSA determined with rTBP and ds29-51, which map downstream of the DPE motif, supports the binding preference of TBP for this sequence (Fig. 7b). Notably, the formation of complexes of reduced mobility between rTBP and the dsM1 oligo pair also indicated the binding preference of TBP for the DPE and DPE downstream sequences, particularly those between nucleotides 38 and 50 of Pr77. This preference was also supported by the finding that no bands of reduced motility were observed in EMSA performed with TBP and an oligo pair (dsM2) that mutated this sequence (Fig. 7a). In addition, the observation of complex formation as a result of TBP and ds ds52-77 oligo pair binding corroborated the importance of the DPE downstream sequence as a target site for TBP binding. The affinity of TBP to the different regions of the Pr77 sequence was also corroborated by an EMSA competition approach in which the ability of each oligo pair under study to displace the complex formed by rTBP and ^32^P-dsPr77 sequence was evaluated. As expected, the rTBP-dsPr77 binding complex could not be displaced by important amounts of ds1-24, ds12-33, ds29-51, ds52-77 and dsM2 (from 5- to 100-fold) used as binding competitors (Additional file 2: Figure S2). However, displacement of 50% of the rTBP-dsPr77 binding complex was obtained with approximately 50× the amount of the ds24-51 oligo pair or the dsM1 oligo pair (Additional file 2: Figure S2).

After that step, due to the involvement of each of these sequences located within Pr77 as binding sites for rTBP, a similar EMSA was performed by incubating the different oligo pairs previously utilized and nuclear proteins of the parasite. As observed in Fig. 7c, several bands of reduced mobility were observed as a result of DNA–protein complex formation when ds24-51, ds52-77, ds29-51, dsM1 and dsM2 and nuclear proteins were incubated. In addition, a less abundant single complex was detected when the radiolabel probes employed were 1–24 and 12–33 oligo pairs (Fig. 7c). Despite the fact that in EMSA carried out with nuclear proteins of the parasite, proteins other than TBP are expected to bind to the ^32^P-radiolabelled oligonucleotide pairs, these results are consistent with those observed in Fig. 7b due to the formation of shifted bands when the oligo pairs were incubated with rTBP were also observed when nuclear proteins of the parasite were mixed with these radiolabelled oligo pairs. Taken together, these results suggested that TBP is a nuclear protein that directly binds along the Pr77 sequence, with the strongest binding occurring at the sequences that bear the DPE and its downstream sequence.

### *T. cruzi* TBP and SNAP50 are part of a protein complex that binds to Pr77

To confirm that TBP and SNAP50 nuclear proteins of the parasite are present in the shifted complexes formed between the parasite nuclear proteins and Pr77 DNA promoter sequence shown in Fig. 7c, an EMSA-crosslinking assay coupled to an immunoblot detection system was performed using nuclear proteins of the parasite and ^32^P-dsPr77 sequence together to the specific monoclonal antibodies directed against rTBP and rSNAP50 proteins.

**Fig. 8:**
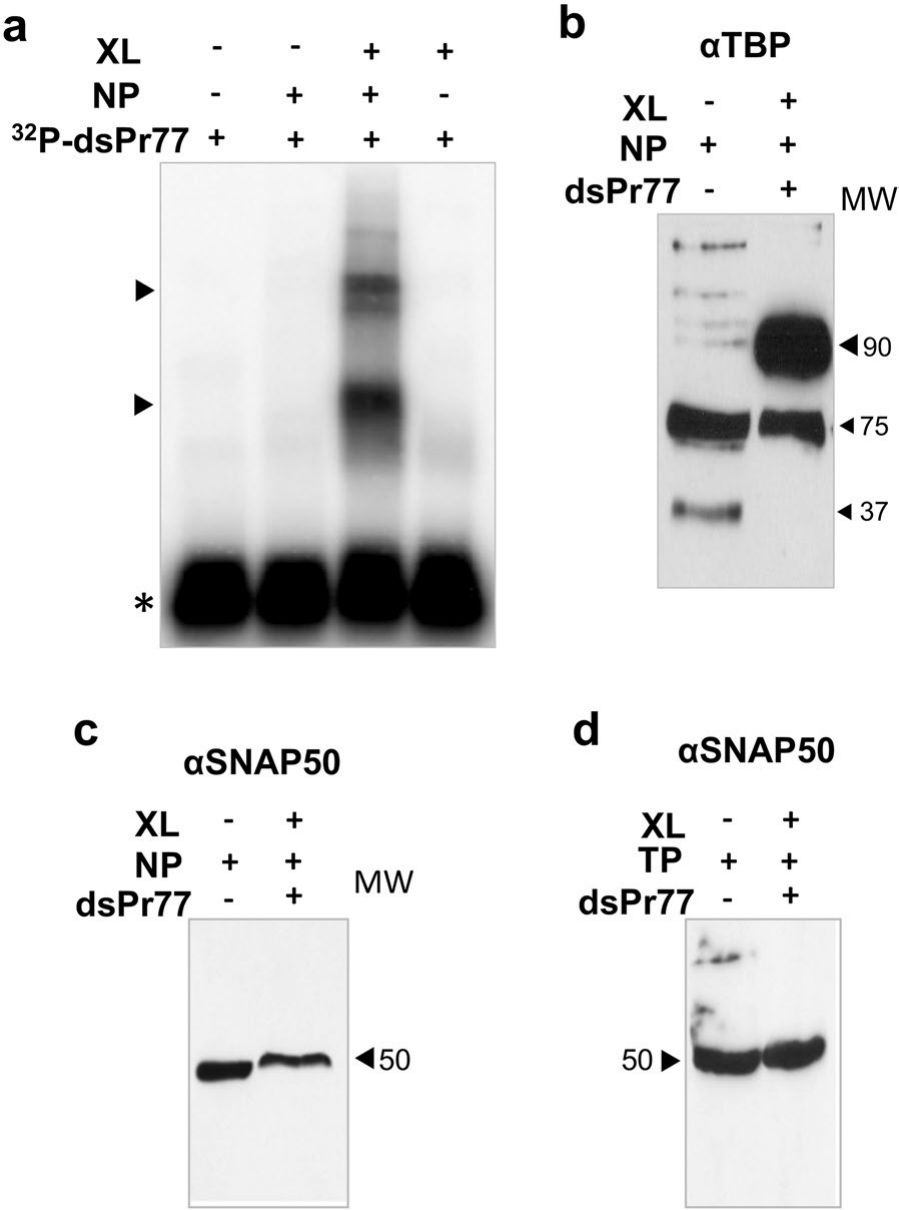
Identification of the nuclear TBP and SNAP50 among the nuclear proteins of the parasite that specifically bind to the Pr77 sequence. **a** The binding reaction was performed using 100,000 cpm of ^32^P-dsPr77 and 3 μg of nuclear proteins (NP) of the parasite by incubation at 37 °C for 30 min. The same reaction was subjected to UV-crosslinking (XL+). The same amount of ^32^P-dsPr77 subjected (+) or not (−) to UV crosslinking (XL) was included as a control. Reactions were loaded on 10% SDS-PAGE denaturing gels, transferred to PVDF membranes and products visualized by PhosphorImager exposure. The black arrowhead indicates the formed complexes of reduced mobility, and the asterisks indicate the electrophoretic mobility of the nucleic acid-free form. **b** In total, 8 pmol (~ 400 ng) of cold dsPr77 probe was incubated with 60 µg of parasite nuclear proteins, and the binding reaction was subjected to UV-crosslinking (XL). Fifty micrograms of nuclear protein from the parasite was loaded as a control. Products were resolved by 10% denaturing SDS-PAGE and transferred to PVDF membranes for subsequent western blot analysis using anti-TBP antibody (dil. 1:150). **c**, **d** In total, 8 pmol (~ 400 ng) of cold dsPr77 probe was incubated with 60 µg of parasite nuclear proteins (**c**) or 180 µg of total proteins (**d**) of *T. cruzi,* and the binding reaction was subjected to UV-crosslinking (XL). The same amount of nuclear (**c**) or total proteins (**d**) of the parasite was loaded as a control. Products were resolved by 10% denaturing SDS-PAGE and transferred to PVDF membranes for subsequent western blot analysis using anti-SNAP50 antibody (dil. 1:5000). The black arrowheads in **b**–**d** indicate the identified products and their molecular weight in kDa

First, analysis showed that the complex formed between the radiolabelled ^32^P-dsPr77 probe and parasite nuclear protein extract stabilized by UV-crosslinked could be visualized after being electrophoresed in SDS-PAGE, transferred to PVDF membrane and exposed to autoradiography. For this purpose, radiolabelled ^32^P-dsPr77 probe and parasite nuclear protein extract were subjected or not to UV-crosslinking to stabilize DNA–protein interactions. The heat-denatured reactions were subsequently resolved in SDS-PAGE denaturing gels and the formed DNA–protein complexes transferred to PVDF membrane for subsequent visualization after autoradiography exposure. As observed in Fig. 8a, the formed complexes that had been previously detected as a result of ^32^P-dsPr77-nuclear protein binding were not detected in the absence of UV crosslinking under denatured conditions (Fig. 8a, NP+, XL−). However, the same reaction subjected to UV-crosslinking showed that the bound complexes formed in the reaction had been sufficiently stabilized by UV-crosslinking that were not dissociated by the resolution of the binding products in an SDS-PAGE denaturing gel, as the two expected shifted complexes were detected (Fig. 8a, lane NP+, XL+). These complexes were shown to be a result of dsPr77 and parasite nuclear protein binding and not a consequence of a structured conformation of the ^32^P-dsPr77 crosslinked probe, as these complexes were not detected when the ^32^P-dsPr77 crosslinked probe alone, employed as a control, was electrophoresed in the denaturing gel (Fig. 8a, NP−, XL+). Next, we proceeded to resolve in denaturing SDS-PAGE a homologous heat-denatured crosslinked binding reactions containing cold dsPr77 probe incubated with parasite nuclear proteins (Fig. 8b, c, lanes NP+, dsPr77+, XL+) together to nuclear proteins of the parasite (Fig. 8b, c, lanes NP+, dsPr77−, XL−), which were included as controls. Gels were transferred to PVDF membranes and analysed by western blotting employing MoAbs directed against rTBP (Fig. 8b) and rSNAP50 (Fig. 8c) proteins to detect their presence in the binding reactions. As expected, in the nuclear protein extracts of the parasite, the anti-TBP and anti-SNAP50 antibodies detected the TBP protein in its monomeric form in addition to other bands consistent with multimeric forms (Fig. 8b, lane NP+, dsPr77−, XL−) as well as a single band of 50 kDa corresponding to the SNAP50 parasite protein (Fig. 8c, lane NP+, dsPr77−, XL−). In addition, the anti-TBP antibody detected the parasite TBP in a shift band of approximately 90 kDa in the X-linked binding reaction (Fig. 8b, lane NP+, dsPr77+, XL+). The immunodetected shifted band of approximately 90 kDa corresponds to the UV-crosslinking stabilized complex formed by the TBP monomer (37 kDa) and the double-stranded Pr77 sequence, which suggests that TBP forms part of the transcription complex that specifically binds to the Pr77 promoter. Interestingly, the TPB 37 kDa monomeric form (observed in Fig. 8b, lane NP+, dsPr77−, XL−) was not detected in the lane containing the crosslinked complex (Fig. 8b, lane NP+, dsPr77+, XL+), which suggests that the monomeric form of TBP had bound to the dsPr77 sequence. In the case of SNAP50 crosslinking reactions (Fig. 8c, lane NP+, dsPr77+, XL+), any band shift was immunodetected by anti-rSNAP50 antibody. These results indicate that in the UV-crosslinked binding reactions, the TBP protein appeared forming part of a complex with a higher molecular size (approximately 90 kDa) than the TBP multimeric form detected in nuclear proteins of the parasite (75 kDa). However, in the case of SNAP50, the size of the specific band detected with the anti-SNAP50 antibody corresponded to that of the soluble monomeric form of the protein. This finding was not related to the nuclear origin of the parasite proteins used in the assay because the same reaction performed with total proteins from the parasite (TP) obtained the same result as the reaction performed with nuclear extracts, detecting a single band of 50 kDa. In addition, the electrophoretic mobility of SNAP50 in the binding reactions carried out with both nuclear (NP) and total (TP) protein extracts was not affected by UV-crosslinking and no stabilized complexes of shift mobility were detected. These data could suggest that the participation of SNAP50 in the complex could be mediated by protein–protein interactions.

**Fig. 9:**
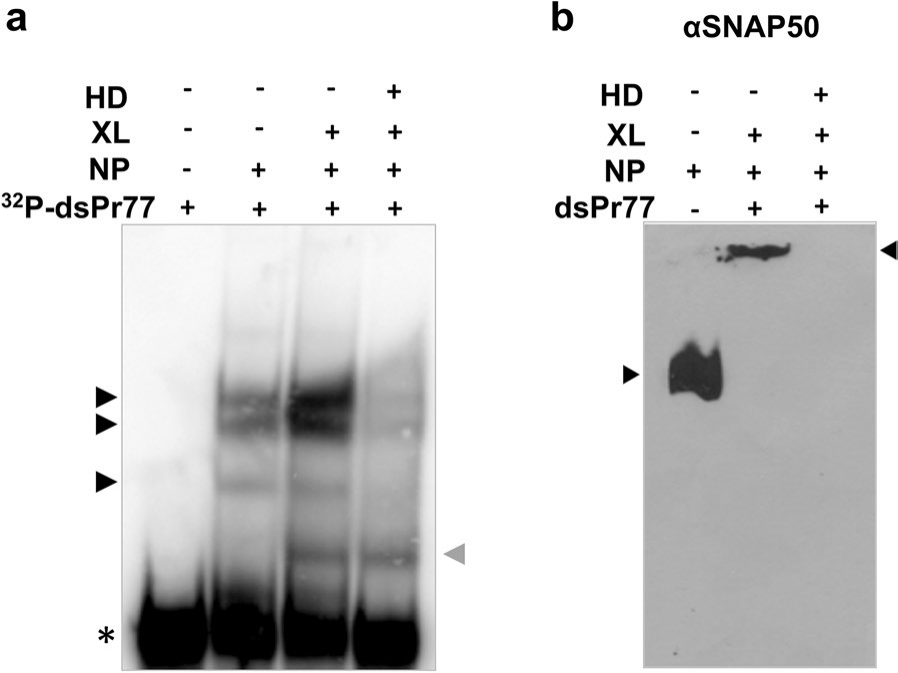
Identification of SNAP50 as part of a heavy protein complex that specifically binds to the Pr77 sequence. **a** The binding reaction of 100,000 ^32^P-dsPr77 and 3 μg of nuclear protein (NP) of the parasite was performed by incubation at 37 °C for 30 min. The same binding reactions were subjected to UV-crosslinking (+) at 3000 W/cm^2^ and 254 nm and to heat denaturing (HD+) by heating at 95 °C for 5 min. A total of 100,000 cpm ^32^P-dsPr77 probe was included as a control. Products were electrophoresed on a 6% polyacrylamide native gel, transferred to PVDF membranes and visualized by PhosphorImager exposure. The black arrowheads indicate the formed complexes of reduced motility, and the asterisk indicates the electrophoretic mobility of the ^32^P-dsPr77 free conformation. **b** The binding reaction was performed by incubating 8 pmol (~ 400 ng) of cold dsPr77 and 60 μg of nuclear protein (NP) of *T. cruzi* at 37 °C for 30 min. The same reactions were subjected to UV-crosslinking (+) and heat denaturing (HD+) by heating at 95 °C for 5 min. Fifty micrograms of nuclear protein (NP) of *T. cruzi* was included as a control. Products were electrophoresed on a 6% polyacrylamide native gel, transferred to PVDF membranes and analysed by western blotting using an anti-SNAP50 specific antibody (dil. 1:5000)

To further analyse whether the binding of SNAP50 protein to the Pr77-transcriptional complex was mediated through protein–protein interactions, the complexes formed as a result of binding between the ^32^P-dsPr77 sequence and nuclear proteins of the parasite were resolved in native acrylamide gels (Fig. 9a, NP+, ^32^P-dsPr77+) together with the same reaction subjected to UV crosslinking (Fig. 9a, NP+, ^32^P-dsPr77+, XL+) and the free probe as a control (Fig. 9a ^32^P-dsPr77+). In addition, the UV-crosslinked binding reaction was also heat-denatured (HD) prior to loading into the native gel (Fig. 9a, NP+, ^32^P-dsPr77+, XL+, HD+). The obtained data allowed us to visualize the formation of at least three complexes of reduced motility (black arrowheads) as a result of complex formation between dsPr77 and nuclear proteins of the parasite (Fig. 9a, NP+, ^32^P-dsPr77+). UV crosslinking of the reaction stabilizes the complexes and leads to observe an increase in the abundance of the binding complexes together with the formation of a fourth complex of faster mobility (Fig. 9a, NP+, ^32^P-dsPr77+, XL+), which is the unique complex (grey arrowhead,) maintained following heat denaturing of the reaction (Fig. 9a, NP+, ^32^P-dsPr77+, XL+, HD+). When the same reactions (NP+, dsPr77+, XL+) were loaded into a native gel together to parasite nuclear proteins employed as a control (Fig. 9b, lane NP+), transferred to a PVDF membrane and analysed by western blotting using the anti-SNAP50 antibody, a SNAP50-containing complex of high molecular weight was observed (Fig. 9b, NP+, dsPr77+, XL+). Heat denaturing of the formed complex induced decoupling of the SNAP50 protein from the complex, as it was not detected (Fig. 9b, NP+, dsPr77+, XL+, HD+).

## Discussion

Trypanosomatids exhibit a scarcity of RNAPII promoters with only two promoter sequences characterised to date, the spliced leader (SL) [39] and Pr77 from L1Tc retrotransposon [14]. However, biochemical characteristics of the transcription factors and DNA motifs that mediate the functions of Pr77-driven transcription have not been determined. We previously reported that *T. cruzi* nuclear proteins specifically bind to the Pr77 promoter, particularly to the DPE motif sequence (CGTG), which plays an important role in L1Tc transcription, suggesting that this sequence acts as a docking site for parasite nuclear factors [15]. This promoter sequence, known as Pr77-hallmark, is also widely distributed in the genome of other trypanosomatids and could therefore play an important role in gene regulation [17].

The present study helps to elucidate Pr77-driven transcription through the identification of TBP and SNAP50 *Trypanosoma cruzi* proteins as Pr77-binding factors as well as determine the preferable DNA–protein interactions and possible protein–protein interactions that take place in this process. Thus, we were able to identify TBP and SNAP50 proteins attached to the Pr77 promoter sequence by using a Dynabeads protein capture assay coupled to western blotting. Thus, TBP and SNAP50 are part of the protein complex that recognizes and specifically interacts with Pr77. Using the same experimental approach, both TBP and SNAP50 proteins were also shown to specifically bind to the SL sequence and not to other sequences such as IL6. Interestingly, the monomeric conformation of TBP (~ 37 kDa) was solely detected in the eluted fractions (E) resulting from the binding reactions of proteins of the parasite and Pr77 and SL probes, although in the flow-through fractions (FT) as in the parasite´ protein extracts, both monomeric and dimeric conformations were detected (~ 37 and ~ 75 kDa, respectively). These data are consistent with previous studies that indicate that TBP predominantly self-associates into dimers through the conserved carboxyl-terminal domain of TBP, and although the TBP dimers do not bind DNA, they must dissociate into monomers before stably binding to the TATA box [40, 41]. These data may suggest that the monomeric fraction of TBP is involved in binding to Pr77 and SL DNA during transcription. This involvement is consistent with results obtained for human and yeast TBP reporting that the recruitment of TBP to promoters is negatively autoregulated by its own dimerization by blocking TBP’s DNA binding sites [40, 41]. Consequently, it has been stated that since TBP binding to TATA represents an early step in transcription complex assembly; the possibility exists that controlling the kinetics of dimer to monomer conversion may, in effect, control transcription complex assembly and initiation [41].

Immunofluorescence microscopy shows that TBP and SNAP50 proteins colocalize in *T. cruzi* epimastigotes in a nuclear major spot. To our knowledge this is the first description of colocalization of TBP and SNAP50 by IFI in trypanosomatids. This result indicates that these two proteins might be very close in the nucleus, suggesting a shared functional role related to Pr77 promoter activity and transcriptional factor characteristics. These data support previous findings in *T. brucei* describing a tight interaction among TBP (or TRF4), TbSNAPc and TFIIA trypanosomal proteins which bind specifically to the SL RNA gene promoter upstream sequence element and are essential for SL RNA gene transcription *in vitro* [22, 23]. This process has also been proposed in *T. cruzi*, although no experimental data supporting this possibility have been reported [31]. Colocalization of TBP and SNAP50 in a single spot in *T. cruzi* epimastigotes is also consistent with the localization of *T. cruzi* RNA polymerase II, the largest subunit which concentrates in a central area in the nucleus near the parasite nucleolus at the genomic SL RNA loci in which there is a high transcriptional activity [42]. The spliced leader-associated RNA polymerase II localization is dependent on the cell transcriptional state and is dispersed when transcription is blocked by α-amanitin and actinomycin D and also in the trypomastigote form where the transcription is diminished [42]. Given the high concentration of pol II at foci in the nucleus, it is possible that other genes, in particular protein coding genes in divergent gene clusters, might also be transcribed by the polymerase in these SL RNA transcription foci [43]. The interspersed character in the genomes of the retrotransposons bearing the Pr77-hallmark would lead to a scattered and not detectable faint signal by IFI or, alternatively, would require that chromosomes be dragged through a pol II focus in a transcription factory-like manner as has been proposed to occur for other genes [43]. The latter would be in accordance with the known fact that transcription in trypanosomatids begins mainly in the Strand Switch Regions and Pr77 bearing retrotransposons, despite being in many chromosomes and being scattered through them, and would be organized so that they are close to the transcription initiation complexes.

TBP protein specifically and directly binds to the Pr77 sequence, exhibiting a strong binding affinity in a positive cooperative manner (Fig. 4a). In contrast, the rSNAP50 protein exhibits a weak interaction with the Pr77 promoter sequence, which is only detected when the Pr77-SNAP50 interaction is stabilized by UV-crosslinking. This suggests that Pr77-SNAP50 interaction requires the participation of other factors, such as some other SNAPc component and/or other cellular proteins that enable SNAP50 participation in the complex. In trypanosomes, SL transcription takes place with the participation of the SNAPc complex, which is composed of three polypeptides measuring 57, 46 and 36 kDa [24, 44]. The 57-kDa polypeptide from *L. seymouri* shows 43% sequence similarity (23% identity) with human SNAP50 and copurifies with 46- and 36-kDa polypeptides and the SL promoter [24, 44]. This fact is consistent with the Dynabeads protein capture data reported in this study, where TBP and SNAP50 show specific binding ability for both Pr77 and SL DNA sequences.

Analysis of the binding kinetics between Pr77 and TBP supports that TBP binds with high specificity to the Pr77 sequence. Thus, EMSA competition assays indicate that the highest displacement of the rTBP-dsPr77 binding complex occurs when Pr77 dsDNA is utilized as a competitor. A large amount of other DNA competitors, such as APT and poly (dI-dC), is required to displace the referred binding complex. The EC50 values obtained in the competition assays indicate that the dsPr77 competitor yields approximately 30 times greater effectiveness in the displacement of the rTBP-^32^P-dsPr77 complex than the APT mixture and 60 times greater effectiveness than the poly (dI-dC) competitor, suggesting an important affinity of rTBP to the Pr77 DNA sequence. TBP binds to the Pr77 sequence as double-stranded and single-stranded nucleic acids and to the sense and antisense Pr77 DNA strands and does not exhibit any binding preference for the structural or conformational status of Pr77 DNA since denaturing the highly structured sequence of dsPr77 did not prevent the binding between Pr77 and TBP. Thus, EMSA performed using both native and denatured Pr77 DNA probes showed that TBP is capable of binding to the Pr77 promoter sequence, regardless of structural and/or conformational issues. Taken together, these results suggest that TBP binding specificity to Pr77 may be attributable to the nucleotide composition of Pr77 and not to structural criteria. In contrast, although recruitment of trypanosomatid TBP to the SL RNA gene promoter has been demonstrated for *Leishmania tarentolae* [30] and is required for SL RNA transcription in *T. brucei*, a direct interaction between TBP and the SL RNA gene promoter DNA was not detected, as observed from the EMSAs [31].

The data obtained using DNA oligo pairs covering the entire Pr77 sequence indicate the existence of several TBP binding sites in the Pr77 nucleotide sequence since formation of different complexes of reduced mobility were observed in the EMSA resolved in native gels. In the case of the oligo pairs comprising the DPE motif and the sequence located downstream of DPE, more than one oligonucleotide pair-rTBP/nuclear protein complexes were observed. This fact was taken as an indication of the different conformations that adopt the formed complexes which depend on the nucleotide composition and the length of each oligonucleotide. The formation of more than one complex may also correspond to an intermediate or transient binding state of a particular complex, which may well be modulated by the strong binding affinity for Pr77 that TBP exhibits in a positive cooperative manner which implies that binding of one molecule of TBP to Pr77 sequence will favour binding of additional TBP molecules to the same sequence. In particular, the regions comprising the DPE motif (CGTG; nucleotides + 25 to + 28) together to the sequence located downstream of DPE (+ 29 to + 51/+ 77) of Pr77 were shown to be important binding sites of TBP and parasite nuclear factors. These results are consistent with the result of EMSA competition assays (Additional file 2: Figure S2) and corroborate the binding affinity of TBP to the abovementioned regions of Pr77 and to the reported preference of *T. cruzi* TBP for C/G-rich DNA sequences in vitro [45]. Although our experimental results show that the + 24 to + 51 region within the Pr77 sequence seems to be the strongest binding region, we cannot rule out the possibility that there may be other regions in the first half of the Pr77 sequence (+ 1 to + 23) serving as *in vivo* binding sites for other parasite nuclear factors. The strong binding of TBP to the + 24 to + 51 region together with its faint binding to the + 12 + 33 region which includes the DPE motif suggests that the DPE motif is not the only the region implicated in transcriptional complex recruitment that mediates Pr77-mediated transcription. These data are consistent with those previously reported in which different point mutations along Pr77 sequence and at the DPE motif abolished the transcriptional capacity of the Pr77 promoter although did not the binding affinity of nuclear proteins [15]. A similar binding pattern to each dsDNA oligo pair to that detected with rTBP was observed using parasite nuclear proteins, although more intensely shifted complexes were detected in most cases. This increased intensity could be attributable to the involvement of the other parasite nuclear proteins with affinity to the Pr77 sequence. In fact, EMSA showed a higher abundance of complexes of reduced mobility when binding assays were performed with dsM2 (DPE-less) and nuclear proteins than when rTBP was used, which again supports the notion that other nuclear proteins specifically bind to Pr77 and cooperate in the recruitment of the Pr77 transcriptional complex.

In light of these results, the data led us to conclude that the DPE motif is an important but not unique binding site of rTBP and certain parasite nuclear proteins to the Pr77 sequence. Thus, there must be nuclear proteins whose binding ability to Pr77 would be mediated through DPE, while others may be in contact with different regions of Pr77, such as those located downstream of DPE. The importance of the DPE motif as a binding site of TBP and other parasite nuclear factors together with its implication in the transcription process may explain the strong degree of conservation in the Pr77-hallmarks from trypanosomatid retrotransposons through evolution [15]. Since both TBP and parasite nuclear proteins show the same pattern of binding to the Pr77 sequence, it appears that the TBP protein participates in the first steps of Pr77 transcriptional complex recruitment.

Based on the finding that the UV-stabilized ^32^P-dsPr77-nuclear protein complexes were maintained following electrophoresis in denaturing conditions, as they were visualized by X-autoradiography exposition, the next aim was to detect the TBP and SNAP50 proteins within the Pr77-transcriptional complex constituted by dsPr77 sequence and nuclear proteins of the parasite. To this end, EMSA was next performed with UV-crosslinked complexes followed by immunoblot detection of the corresponding protein factors using specific antibodies raised against TBP and SNAP50. As observed before, the monomeric (37 kDa) and multimeric (e.g., 75 kDa) conformations of the TBP nuclear protein of the parasite were detected by the anti-TBP antibody. In the binding reaction and, in addition to the dimeric form of the TBP (approximately 75 kDa), the anti-TBP antibody detected a complex with reduced motility (of approximately 90 kDa) resulting from dsPr77 and TBP binding. However, in this binding reaction the monomeric form of TBP was not detected, which suggests that monomeric TBP was involved in the specific binding of TBP to the Pr77 sequence. These findings were consistent with the results of the Dynabeads protein capture in which the monomeric form of the protein is bound to the Pr77 sequence, and also with the results of the EMSA and EMSA competition assays, which indicated specific and direct binding of TBP to the Pr77 sequence. This specific binding followed a cooperative binding model, as the Hill coefficient (*α*_H_) was 1.67. This *α*_*H*_ value > 1 implies that the binding of one molecule of TBP to Pr77 facilitates the binding of subsequent ligands to other Pr77 sites and that the binding of TBP molecules to the different sites on Pr77 does not constitute mutually independent events. All these findings suggest that TBP is a factor that initially binds to Pr77, favouring the recruitment of additional factors related or not related to TBP.

Detection of SNAP50 in the complex constituted by Pr77 and nuclear proteins together with the Dynabead protein capture results indicate that SNAP50 protein also forms part of the Pr77-nuclear protein binding complexes. Analysis of the complexes formed between dsPr77 and nuclear proteins of the parasite resolved in native gels enabled the detection of several complexes of reduced motility. Although these complexes were stabilized, as expected, by UV-crosslinking, some of them were heat-resistant, while others were heat-sensitive, as they were dissociated by heat denaturing under the experimental conditions. The presence of SNAP50 as one of the proteins conforming to the DNA–protein binding complex was corroborated by resolution in native gel of homologous no heat and heat-denatured UV-crosslinked binding reactions followed by immunoblotting employing an anti-SNAP50-specific antibody, which led to the detection of a complex with highly reduced mobility. The presence of SNAP50 in the heavy and heat-sensitive complex conformed by dsPr77 DNA and several nuclear proteins of the parasite together with the weak direct binding observed between SNAP50 and the dsPr77 sequence strongly suggest that the participation of SNAP50 in this complex is mediated through protein–protein interactions with other parasite factors, which may be SNAPc proteins. This finding is consistent with the structure of the trypanosomal SNAPc (tSNAPc), which consists of a trimeric conformer that contains the SNAP50, SNAP42 and SNAP26 proteins [32]. In addition, several studies have shown that *T. brucei* TBP and SNAP50 interact with each other and with SL promoter DNA to form the preinitiation complex, which subsequently recruits the rest of the factors and RNA polymerase II [32].

Taken together, these findings strongly suggest that both the *T. cruzi* TBP and SNAP50 are transcription factors involved in Pr77-driven transcription. To the best of our knowledge, these results are the first to identify transcription factors binding a retrotransposon promoter in trypanosomes. These finding strongly suggests that a preinitiation complex is formed in the Pr77 promoter that may well involve the 27 proteins that have been characterized to form the PIC on the *T. brucei* SL RNA gene promoter. Although further studies are required to elucidate the basic mechanisms required for functional RNA polymerase II-dependent transcription in *Trypanosoma cruzi*, our results shed light on the Pr77-hallmark mediated transcription process of the non-LTR retrotransposons in this organism.

## Conclusions

The limited knowledge of the factors correlated with unusual modes of transcription in trypanosomatids and, particularly in *Trypanosome cruzi*, highlights the need for studies to elucidate how transcriptional processes are mediated in these parasites. In this article, to our knowledge we present the first description of TBP and SNAP50 as transcription factors that specifically bind to the Pr77 promoter of non-LTR retrotransposons of *T. cruzi*. Our findings indicate that the TBP protein binds directly to the Pr77 sequence, in particular to the regions comprising the DPE motif and the downstream DPE sequence, exhibiting strong binding affinity in a positive cooperative manner. The findings also indicate that there is no direct binding of SNAP50 to the Pr77 sequence. In these organisms, studies are needed to understand the influence of the transcriptional capacity of non-LTR retrotransposons on the transcription of polycistrons where these mobile DNA elements are interspersed.

## Supplementary Information

**Additional file 1: Figure S1.** Binding kinetics of rTBP to the dsPr77 sequence by EMSA. A 0.33 nM concentration of ^32^P-labelled dsPr77 was preincubated with increasing concentrations (0.32–6.35 µM) of rTBP at 37 °C for 30 min. Control reactions were performed without protein. Reactions were loaded on 6% native polyacrylamide gels, and quantification was carried out in a PhosphorImager. The results were obtained from three independent experiments, as shown in Fig. 4a (top panel). The average values corresponding to the bound dsDNA fraction were plotted against the protein concentration, as shown in Fig. 4a (bottom panel). The curve corresponds to the best fit of the Hill equation to the experimental data [*R*^2^ (coefficient of determination) = 0.97]. The equation used was as follows: $y = \frac{{B_{\max } \cdot x^{{\alpha_{{\text{H}}} }} }}{{K_{{\text{d}}}^{{\alpha_{{\text{H}}} }} + x^{{\alpha_{{\text{H}}} }} }}$, where ‘*x*’ is the protein concentration and ‘*y*’ is the radiolabelled dsDNA-bound fraction. *K*_d_*,* defined as the protein concentration at which 50% of the dsDNA is bound, is indicated.

**Additional file 2: Figure S2.** Determination of the preferential binding site/s of rTBP and nuclear proteins to the Pr77 sequence. **a** Binding kinetics of rTBP to dsPr77 and double-stranded oligo pairs mapping the Pr77 sequence by EMSA. A 0.33 nM concentration of ^32^P-dsPr77 or each ^32^P-labelled oligo-pair was preincubated with increasing concentrations (0.63, 3.17, 6.35 µM) of rTBP at 37 °C for 30 min. Control reactions were performed without protein. Reactions were loaded on 6% native polyacrylamide gels, and quantification was carried out in a PhosphorImager. **b** Binding specificity of TBP to the Pr77 sequence by EMSA competition. EMSA was carried out after preincubation of 0.125 nM ^32^P-dsPr77 with 1.58 μM rTBP and with different concentrations (as indicated, fold excess ranging from 5 to 500 times) of each cold oligo pair as DNA competitors. Control reactions were performed in **a** and **b** without protein. Reactions were loaded on 6% native polyacrylamide gels, and the results were visualized in a PhosphorImager. Shifted bands are indicated with black arrowheads, and the radiolabelled free form of each probe is indicated with an asterisk.

**Additional file 3: Table S3.** Sequence of oligonucleotides used in binding assays.

## Data Availability

The datasets supporting the conclusions of this article are available within the article. The DNA coding sequences of the TBP and SNAP50 proteins from *T. cruzi* are available in GenBank under the accession numbers AF465747.1 and XM_803702, respectively.

## Abbreviations

LINE: Long interspersed nuclear element
SINE: Short interspersed nuclear element
SIDER: Short interspersed degenerated retroposons
DIRE: Degenerate *ingi*/L1Tc-related element
TBP: TATA-binding protein
SNAP: Small nuclear RNA-activating protein
EMSA: Electrophoretic mobility shift assay
DPE: Downstream promoter element
RNAPII and III: RNA polymerase II and III
SL: Spliced leader
LIT: Liver infusion tryptose medium
RP: Recombinant protein
TP: Total protein extracts
NP: Nuclear protein extracts
CP: Cytoplasmic protein extracts
IPTG: Isopropyl β-d-thiogalactoside
PVDF: Polyvinylidene fluoride
DTT: Dithiothreitol
PMSF: Phenylmethanesulfonyl fluoride
BSA: Bovine serum albumin
SELEX: Systematic evolution of ligands by exponential enrichment
FP: Free probe
DMSO: Dimethyl sulfoxide
MoAb: Monoclonal antibody
UV-XL: Ultraviolet crosslinking
APT: Aptamer
HD: Heat denaturing

## References

[1] WHO. Chagas disease (*Trypanosoma cruzi*)*.* 2020. https://www.who.int/news-room/fact-sheets/detail/chagas-disease-(american-trypanosomiasis).

[2] WHO. Sleeping sickness (*Trypanosoma brucei*). 2020. https://www.who.int/en/news-room/fact-sheets/detail/trypanosomiasis-human-african-(sleeping-sickness).

[3] WHO. Leishmaniasis (*Leishmania* spp.). 2020. https://www.who.int/news-room/fact-sheets/detail/leishmaniasis.

[4] Clayton C (2019). Regulation of gene expression in trypanosomatids: living with polycistronic transcription. Open Biol.

[5] Bringaud F, Biteau N, Zuiderwijk E, Berriman M, El-Sayed NM, Ghedin E (2004). The ingi and RIME non-LTR retrotransposons are not randomly distributed in the genome of *Trypanosoma brucei*. Mol Biol Evol.

[6] Bringaud F, Bartholomeu DC, Blandin G, Delcher A, Baltz T, El-Sayed NMA (2006). The *Trypanosoma cruzi* L1Tc and NARTc non-LTR retrotransposons show relative site specificity for insertion. Mol Biol Evol.

[7] Bringaud F, Müller M, Cerqueira GC, Smith M, Rochette A, El-Sayed NMA (2007). Members of a large retroposon family are determinants of post-transcriptional gene expression in *Leishmania*. PLoS Pathog.

[8] Bringaud F, Ghedin E, Blandin G, Bartholomeu DC, Caler E, Levin MJ (2006). Evolution of non-LTR retrotransposons in the trypanosomatid genomes: *Leishmania major* has lost the active elements. Mol Biochem Parasitol.

[9] Olivares M, Thomas MC, Alonso C, López MC (1999). The L1Tc, long interspersed nucleotide element from *Trypanosoma cruzi*, encodes a protein with 3′-phosphatase and 3′-phosphodiesterase enzymatic activities. J Biol Chem.

[10] García-Pérez JL, González CI, Thomas MC, Olivares M, López MC (2003). Characterization of reverse transcriptase activity of the L1Tc retroelement from *Trypanosoma cruzi*. Cell Mol Life Sci.

[11] Heras SR, López MC, García-Pérez JL, Martin SL, Thomas MC (2005). The L1Tc C-terminal domain from *Trypanosoma cruzi* non-long terminal repeat retrotransposon codes for a protein that bears two C_2_H_2_ zinc finger motifs and is endowed with nucleic acid chaperone activity. Mol Cell Biol.

[12] Heras SR, Thomas MC, Macias F, Patarroyo ME, Alonso C, López MC (2009). Nucleic-acid-binding properties of the C2-L1Tc nucleic acid chaperone encoded by L1Tc retrotransposon. Biochem J.

[13] Thomas MC, Macias F, Alonso C, López MC (2010). The biology and evolution of transposable elements in parasites. Trends Parasitol.

[14] Heras SR, López MC, Olivares M, Thomas MC (2007). The L1Tc non-LTR retrotransposon of *Trypanosoma cruzi* contains an internal RNA-pol II-dependent promoter that strongly activates gene transcription and generates unspliced transcripts. Nucleic Acids Res.

[15] Macías F, López MC, Thomas MC (2016). The trypanosomatid Pr77-hallmark contains a downstream core promoter element essential for transcription activity of the *Trypanosoma cruzi* L1Tc retrotransposon. BMC Genom.

[16] Bringaud F, García-Pérez JL, Heras SR, Ghedin E, El-Sayed NM, Andersson B (2002). Identification of non-autonomous non-LTR retrotransposons in the genome of *Trypanosoma cruzi*. Mol Biochem Parasitol.

[17] Sánchez-Luque FJ, López MC, Macias F, Alonso C, Thomas MC (2012). Pr77 and L1TcRz: a dual system within the 5′-end of L1Tc retrotransposon, internal promoter and HDV-like ribozyme. Mob Genet Elem.

[18] Sánchez-Luque FJ, López MC, MacIas F, Alonso C, Thomas MC (2011). Identification of an hepatitis delta virus-like ribozyme at the mRNA 5′-end of the L1Tc retrotransposon from *Trypanosoma cruzi*. Nucleic Acids Res.

[19] Sánchez-Luque FJ, López MC, Carreira PE, Alonso C, Thomas MC (2014). The wide expansion of hepatitis delta virus-like ribozymes throughout trypanosomatid genomes is linked to the spreading of L1Tc/ingi clade mobile elements. BMC Genom.

[20] Ruan J, Arhin GK, Ullu E, Tschudi C (2004). Functional characterization of a *Trypanosoma brucei* TATA-binding protein-related factor points to a universal regulator of transcription in trypanosomes. Mol Cell Biol.

[21] Schimanski B, Laufer G, Gontcharova L, Günzl A (2004). The *Trypanosoma brucei* spliced leader RNA and rRNA gene promoters have interchangeable TbSNAP50-binding elements. Nucleic Acids Res.

[22] Das A, Zhang Q, Palenchar JB, Chatterjee B, Cross GAM, Bellofatto V (2005). Trypanosomal TBP functions with the multisubunit transcription factor tSNAP to direct spliced-leader RNA gene expression. Mol Cell Biol.

[23] Schimanski B (2005). Characterization of a multisubunit transcription factor complex essential for spliced-leader RNA gene transcription in *Trypanosoma brucei*. Mol Cell Biol.

[24] Das A, Bellofatto V (2003). RNA polymerase II-dependent transcription in trypanosomes is associated with a SNAP complex-like transcription factor. Proc Natl Acad Sci USA.

[25] Lecordier L, Devaux S, Uzureau P, Dierick JF, Walgraffe D, Poelvoorde P (2007). Characterization of a TFIIH homologue from *Trypanosoma brucei*. Mol Microbiol.

[26] Lee JH, Nguyen TN, Schimanski B, Günzl A (2007). Spliced leader RNA gene transcription in *Trypanosoma brucei* requires transcription factor TFIIH. Eukaryot Cell.

[27] Lee JH, Jung HS, Günzl A (2009). Transcriptionally active TFIIH of the early-diverged eukaryote *Trypanosoma brucei* harbors two novel core subunits but not a cyclin-activating kinase complex. Nucleic Acids Res.

[28] Palenchar JB, Liu W, Palenchar PM, Bellofatto V (2006). A divergent transcription factor TFIIB in trypanosomes is required for RNA polymerase II-dependent spliced leader RNA transcription and cell viability. Eukaryot Cell.

[29] Schimanski B, Brandenburg J, Nguyen TN, Caimano MJ, Günzl A (2006). A TFIIB-like protein is indispensable for spliced leader RNA gene transcription in *Trypanosoma brucei*. Nucleic Acids Res.

[30] Thomas S, Yu MC, Sturm NR, Campbell DA (2006). A non-universal transcription factor? The *Leishmania tarentolae* TATA box-binding protein LtTBP associates with a subset of promoters. Int J Parasitol.

[31] Cribb P, Serra E (2009). One- and two-hybrid analysis of the interactions between components of the *Trypanosoma cruzi* spliced leader RNA gene promoter binding complex. Int J Parasitol.

[32] Das A, Banday M, Bellofatto V (2008). RNA polymerase transcription machinery in trypanosomes. Eukaryot Cell.

[33] Thomas S, Green A, Sturm NR, Campbell DA, Myler PJ (2009). Histone acetylations mark origins of polycistronic transcription in *Leishmania majo*r. BMC Genom.

[34] Morón B, Cebolla Á, Manyani H, Álvarez-Maqueda M, Megías M, Thomas MDC (2008). Sensitive detection of cereal fractions that are toxic to celiac disease patients by using monoclonal antibodies to a main immunogenic wheat peptide. Am J Clin Nutr.

[35] Sambrook J, Fritsch EF, Maniatis T (1989). Molecular cloning: a laboratory manual.

[36] Marañón C, Thomas MC, Puerta C, Alonso C, López MC (2000). The stability and maturation of the H2A histone mRNAs from *Trypanosoma cruzi* are implicated in their post-transcriptional regulation. Biochim Biophys Acta Gene Struct Expr.

[37] Gómez Pérez V, García-Hernandez R, Corpas-López V, Tomás AM, Martín-Sanchez J, Castanys S (2016). Decreased antimony uptake and overexpression of genes of thiol metabolism are associated with drug resistance in a canine isolate of Leishmania infantum. Int J Parasitol Drugs Drug Resist.

[38] Nunes LR, De Carvalho MRC, Buck GA (1997). *Trypanosoma cruzi* strains partition into two groups based on the structure and function of the spliced leader RNA and rRNA gene promoters. Mol Biochem Parasitol.

[39] Campbell DA, Thomas S, Sturm NR (2003). Transcription in kinetoplastid protozoa: why be normal?. Microbes Infect.

[40] Coleman RA, Taggart AKP, Benjamin LR, Pugh BF (1995). Dimerization of the TATA binding protein. J Biol Chem.

[41] Jackson-Fisher AJ, Chitikila C, Mitra M, Pugh BF (1999). A role for TBP dimerization in preventing unregulated gene expression. Mol Cell.

[42] Dossin FDM, Schenkman S (2005). Actively transcribing RNA polymerase II concentrates on spliced leader genes in the nucleus of *Trypanosoma cruzi*. Eukaryot Cell.

[43] Daniels J-P, Gull K, Wickstead B (2010). Cell biology of the trypanosome genome. Microbiol Mol Biol Rev.

[44] Luo H, Bellofatto V (1997). Characterization of two protein activities that interact at the promoter of the trypanosomatid spliced leader RNA. J Biol Chem.

[45] Cribb P, Esteban L, Trochine A, Girardini J, Serra E (2010). *Trypanosoma cruzi* TBP shows preference for C/G-rich DNA sequences in vitro. Exp Parasitol.

