# Supplementary figures and images for: TBP and SNAP50 transcription factors bind specifically to the Pr77 promoter sequence from trypanosomatid non-LTR retrotransposons
Source: Parasit Vectors. 2021 Jun 9;14:313. doi: 10.1186/s13071-021-04803-5 (PMC8190864; doi:10.1186/s13071-021-04803-5)

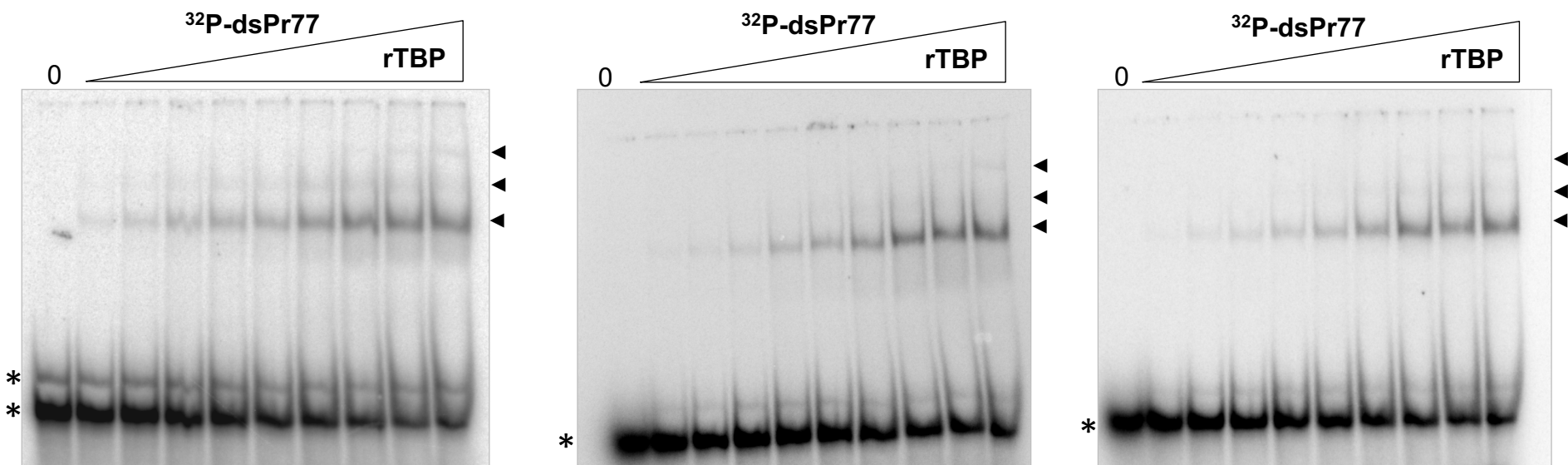

Figure S1

Supplement: Supplementary file 1 — Additional file 1: Figure S1. Binding kinetics of rTBP to the dsPr77 sequence by EMSA. A 0.33 nM concentration of 32P-labelled dsPr77 was preincubated with increasing concentrations (0.32–6.35 µM) of rTBP at 37 °C for 30 min. Control reactions were performed without protein. Reactions were loaded on 6% native polyacrylamide gels, and quantification was carried out in a PhosphorImager. The results were obtained from three independent experiments, as shown in Fig. 4a (top panel). The average values corresponding to the bound dsDNA fraction were plotted against the protein concentration, as shown in Fig. 4a (bottom panel). The curve corresponds to the best fit of the Hill equation to the experimental data [R2 (coefficient of determination) = 0.97]. The equation used was as follows: $y = \frac{{B_{\max } \cdot x^{{\alpha_{{\text{H}}} }} }}{{K_{{\text{d}}}^{{\alpha_{{\text{H}}} }} + x^{{\alpha_{{\text{H}}} }} }}$y=Bmax·xαHKdαH+xαH, where ‘x’ is the protein concentration and ‘y’ is the radiolabelled dsDNA-bound fraction. Kd, defined as the protein concentration at which 50% of the dsDNA is bound, is indicated. [file 13071_2021_4803_MOESM1_ESM.pdf]

**a**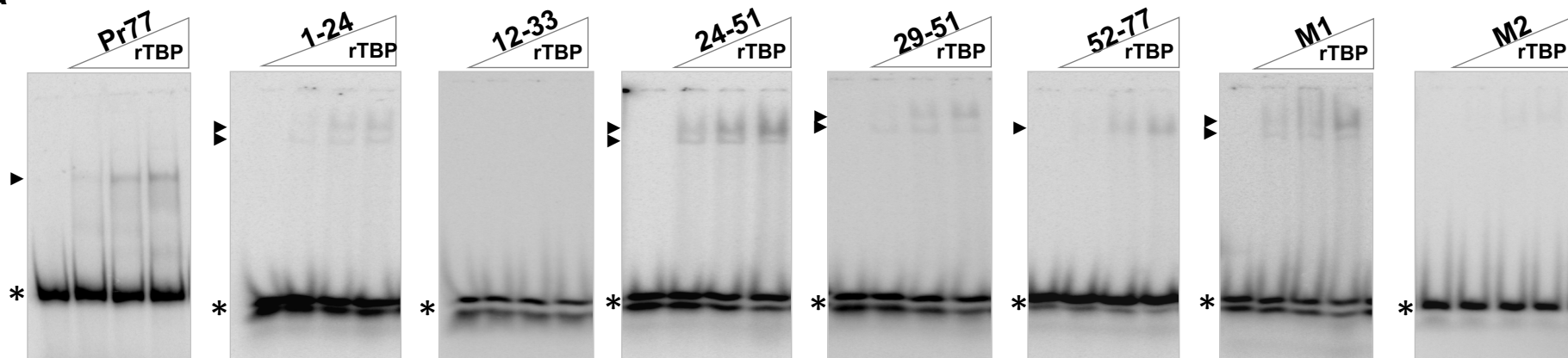**b**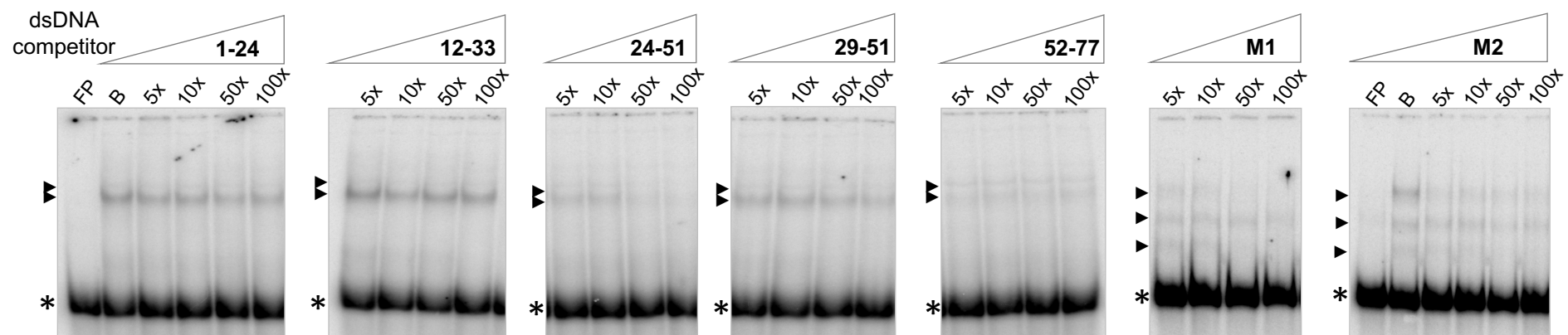

Figure S2

Supplement: Supplementary file 2 — Additional file 2: Figure S2. Determination of the preferential binding site/s of rTBP and nuclear proteins to the Pr77 sequence. a Binding kinetics of rTBP to dsPr77 and double-stranded oligo pairs mapping the Pr77 sequence by EMSA. A 0.33 nM concentration of 32P-dsPr77 or each 32P-labelled oligo-pair was preincubated with increasing concentrations (0.63, 3.17, 6.35 µM) of rTBP at 37 °C for 30 min. Control reactions were performed without protein. Reactions were loaded on 6% native polyacrylamide gels, and quantification was carried out in a PhosphorImager. b Binding specificity of TBP to the Pr77 sequence by EMSA competition. EMSA was carried out after preincubation of 0.125 nM 32P-dsPr77 with 1.58 μM rTBP and with different concentrations (as indicated, fold excess ranging from 5 to 500 times) of each cold oligo pair as DNA competitors. Control reactions were performed in a and b without protein. Reactions were loaded on 6% native polyacrylamide gels, and the results were visualized in a PhosphorImager. Shifted bands are indicated with black arrowheads, and the radiolabelled free form of each probe is indicated with an asterisk. [file 13071_2021_4803_MOESM2_ESM.pdf]
