# Supplementary material for: TBP and SNAP50 transcription factors bind specifically to the Pr77 promoter sequence from trypanosomatid non-LTR retrotransposons
Source: Parasit Vectors. 2021 Jun 9;14:313. doi: 10.1186/s13071-021-04803-5 (PMC8190864; doi:10.1186/s13071-021-04803-5)
Supplement: Supplementary file 3 — Additional file 3: Table S3. Sequence of oligonucleotides used in binding assays. [file 13071_2021_4803_MOESM3_ESM.docx]

**Additional file 3: Table S3.** Sequence of oligonucleotides used in binding assays.

| **Name** | **Sequence (5’⭢3’)** |
| --- | --- |
| Biot-Pr77s | Biot-CCCTGGCTCAGCCGGCCACCTCAACGTGGTGCCAGGGTCTAGTACTCTTTGCTAGAGAGGAAGCTAAGCGCCTGCTG |
| Biot-SLs | Biot-GGGTTTTTGTACACGGTCCCAAGTGCCGCGAAGGACCCCTCATCAAAATTGAAAACCGTTGTGGAACACAACTTCCTTTCAACTAACGCT |
| SLas | AGCGTTAGTTGAAAGGAAGTTGTGTTCCACAACGGTTTTCAATTTTGATGAGGGGTCCTTCGCGGCACTTGGGACCGTGTACAAAAACCC |
| Biot-IL6s | Biot-GGTACATCCTCGACGGCATCTCAGCCCTGAGAAAGGAGACATGTAACAAGAGTAACATGTGTGAAAGCAGCAAAGAGG |
| IL6as | CCTCTTTGCTTTCACACATGTTACTCTTGTTACATGTCTCCTTTCTCAGGGCTGAGATGCCGTCGAGGATGTACC |
| Pr77s | CCCTGGCTCAGCCGGCCACCTCAACGTGGTGCCAGGGTCTAGTACTCTTTGCTAGAGAGGAAGCTAAGCGCCTGCTG |
| Pr77as | CAGCAGGCGCTTAGCTTCCTCTCTAGCAAAGAGTACTAGACCCTGGCACCACGTTGAGGTGGCCGGCTGAGCCAGGG |
| 1-24s | CCCTGGCTCAGCCGGCCACCTCAA |
| 1-24as | TTGAGGTGGCCGGCTGAGCCAGGG |
| 12-33s | CCGGCCACCTCAACGTGGTGCC |
| 12-33as | GGCACCACGTTGAGGTGGCCGG |
| M1s | CAACGTGGTATAGTGGTCTAGTACTC |
| M1as | GAGTACTAGACCACTATACCACGTTG |
| 24-51s | ACGTGGTGCCAGGGTCTAGTACTCTTTG |
| 24-51as | CAAAGAGTACTAGACCCTGGCACCACGT |
| M2s | GGTGCCAGGGGATCGTACTCTTTGC |
| M2as | GCAAAGAGTACGATCCCCTGGCACC |
| 29-51s | GTGCCAGGGTCTAGTACTCTTTG |
| 29-51as | CAAAGAGTACTAGACCCTGGCAC |
| 52-77s | CTAGAGAGGAAGCTAAGCGCCTGCTG |
| 52-77as | CAGCAGGCGCTTAGCTTCCTCTCTAG |
